# Supplementary material for: Identification of Polyphenol Derivatives as Novel SARS-CoV-2 and DENV Non-Nucleoside RdRp Inhibitors
Source: Molecules. 2022 Dec 25;28(1):160. doi: 10.3390/molecules28010160 (PMC9822497; doi:10.3390/molecules28010160)
Supplement: Supplementary file 1 [file molecules-28-00160-s001.zip › molecules-2063545-supplementary.pdf]

**Supporting Information for**

# Identification of Polyphenol Derivatives as novel SARS-CoV-2 and DENV Non-Nucleoside RdRp Inhibitors

Shenghua Gao,[1,2,3,†] Letian Song,[1,2,†] Hongtao Xu,[4*] Antonios Fikatas,[4] Merel Oeyen,[4] Steven De Jonghe,[4] Fabao Zhao,[1,2] Lanlan Jing,[1,2] Dirk Jochmans,[4] Laura Vangeel,[4] Yusen Cheng,[1,2] Johan Neyts,[4] Piet Herdewijn,[5] Dominique Schols,[4*] Peng Zhan,[1, 2*] and Xinyong Liu [1, 2*]

**Corresponding Authors**

*X.L.: e-mail, xinyongl@sdu.edu.cn; Tel, 086-531-88380270;

*P.Z.: e-mail, zhanpeng1982@sdu.edu.cn; Tel, 086-531-88382005;

*H.X.: e-mail, hongtao.xu@kuleuven.be.

*D.S.: e-mail, dominique.schols@kuleuven.be

# Table of Contents

**Experimental section**........................................S3
General Experimental Methods........................................S3
Synthetic Routes........................................S3
Preparation of Intermediates........................................S4
General Procedure for Compounds Except DF-51........................................S8
Spectral Data for Compounds Except DF-51........................................S8
Synthetic Procedure and Spectral Data of DF-51........................................S12
**NMR Spectra of Final Compounds**........................................S13
**MS Spectra of Final Compounds**........................................S23
**HPLC Traces of Representative Compounds**........................................S29
**Full List of In-house Metal Chelator Library Compounds**........................................S30

**Experimental Section**

*General experimental methods.*

All melting points were determined on a micro melting point apparatus (RY-1G, Tianjin TianGuang Optical Instruments). All chemical reagents and reaction solvents were purchased from commercial suppliers. $^{1}$H NMR and $^{13}$C NMR spectra were recorded in DMSO-$d_6$ on a Bruker AV-400 spectrometer, with tetramethyl silane (TMS) as the internal standard. Coupling constants were given in hertz, and chemical shifts were reported in δ values (ppm) from TMS; signals were abbreviated as s (singlet), d (doublet), t (triplet), q (quarter), and m (multiplet). A G1313A Standard LC autosampler (Agilent) was used to collect samples for measurement of mass spectra. All reactions were routinely monitored by thin layer chromatography (TLC) on silica gel GF254. Flash column chromatography was performed on columns packed with silica gel (200-300 mesh), purchased from Qingdao Haiyang Chemical Company. The purities of representative final compounds were tested on an Agilent 1260 HPLC system. HPLC conditions: Agilent ZORBAX, SB-C18 column (250 mm × 4.6 mm × 5 μm). methanol/water with 65:35; flow rate 1.0 mL/min; temperature, 40 °C; injection volume, 10 μL.

**Scheme 1. Synthetic Route to DF-35, DF-36, DF-47, DF-51.**

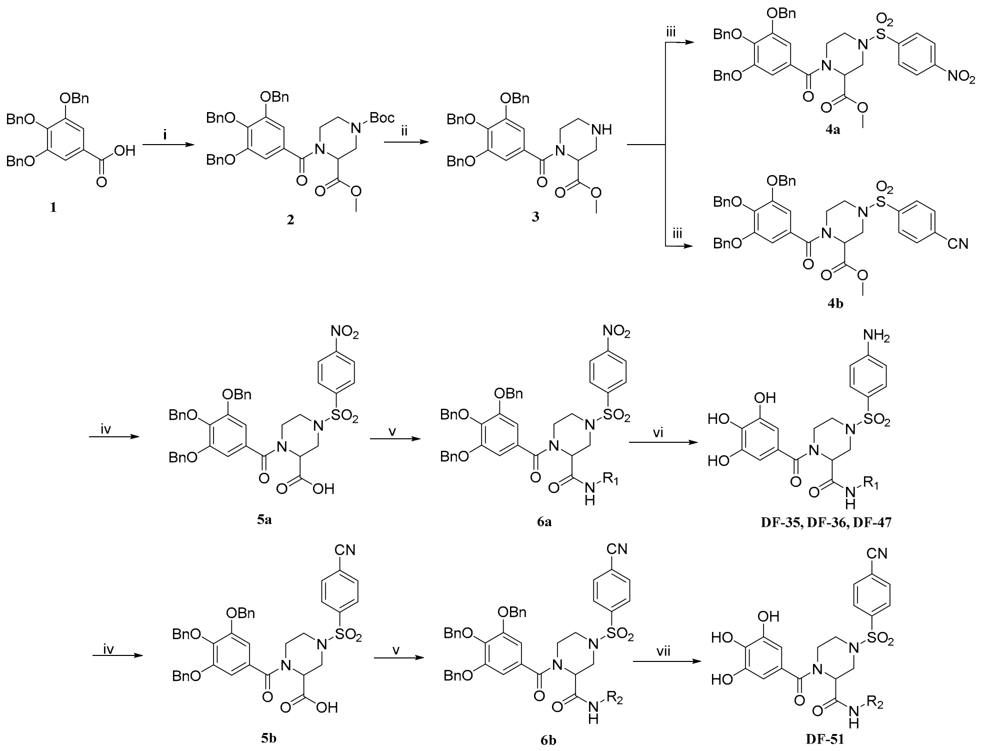


**Reagents and Conditions:** i) 1-(tert-butyl) 2-methyl piperazine-1,2-dicarboxylate, EDCI, HOBt, $Et_3N$, DMF, r.t., 12 h; ii) TFA/DCM, r.t., 4 h; iii) $RSO_2Cl$, $Et_3N$, DCM, r.t., 12 h; iv) LiOH, $H_2O$; v) HATU, DIEA, 0 ºC-r.t.; vi) $H_2$, Pd/C, MeOH/DCM, rt, 12 h; vii) $BCl_3$, DCM, -45 °C.

**Scheme 2. Synthetic Route to DF-57, DF-63, DF-64, DF-67, DF-68, DF-69, DF-71.**

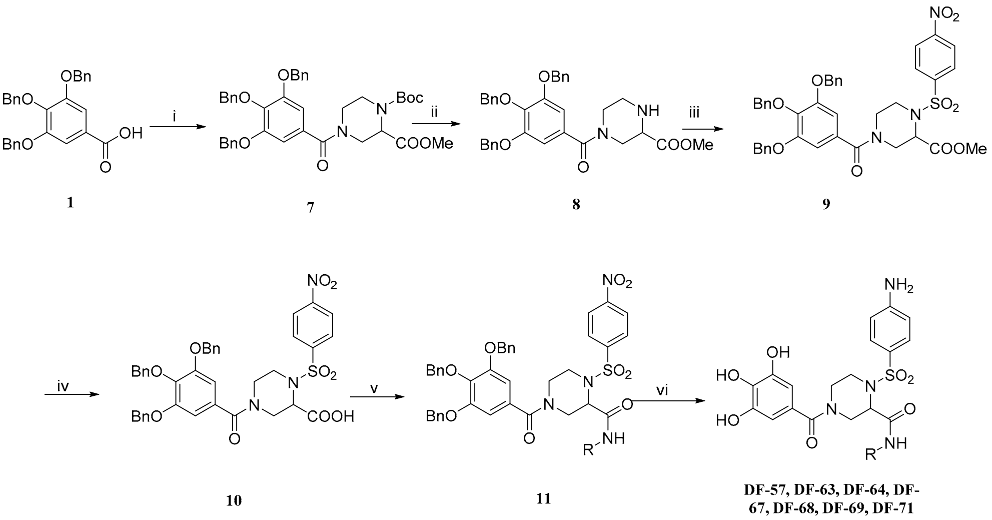


**Reagents and Conditions:** i) 1-(tert-butyl) 3-methyl piperazine-1,3-dicarboxylate, EDCI, HOBt, $Et_3N$, DMF, r.t., 12 h; ii) TFA/DCM, r.t., 4 h; iii) $RSO_2Cl$, $Et_3N$, DCM, r.t., 12 h; iv) LiOH, $H_2O$; v) HATU, DIEA, 0 ºC- r.t.; vi) $H_2$, Pd/C, MeOH/DCM, r.t.,

12 h;

**The general procedure for intermediate 2 and 7**:

A 250 mL round-bottom flask was charged with 3,4,5-tris(benzyloxy)benzoic acid (**1**, 2.018 g, 4.52 mmol) and dissolved with 50 mL *N,N*-dimethylformamide (DMF) under ice bath, followed by 1-ethyl-3-dimethylpropanylcarbodiimide hydrochloride (EDCI, 0.93 g, 4.89 mmol), 1-hydroxybenzotriazole (HOBt, 0.64 g, 4.97 mmol), and triethylamine (1.25 mL, 10.06 mmol). After the mixture was stirred over 1 h, the corresponding *N*-Boc piperazine carboxylic acid methylate (1.2 eq) was added to the mixture, and then stirred at room temperature for 12 h (monitored by TLC). 150 mL water was added to quench the reaction when the reaction was finished. Extract the water phase with EtOAc (3×60 mL). The organic layer was separated, washed with saturated brine (3×20 mL) and dried over anhydrous sodium sulfate in proper sequence. The filtrate was concentrated and the residue was purified by silica gel column chromatography to give the intermediate **2** or **7**.

***1-(3,4,5-tris(benzyloxy)benzoyl)-4-Boc piperazine-2-carboxylic acid methylate (2)***

White solid, 75% yield, melting point 101-104°C. $^1$H NMR (400 MHz, DMSO-$d_6$) δ 7.42 (dq, *J* = 7.1, 1.1 Hz, 6H), 7.35 (t, *J* = 6.7 Hz, 6H), 7.39 – 7.25 (m, 3H), 6.95 (s, 2H), 5.14 – 5.05 (m, 6H), 4.78 (t, *J* = 5.2 Hz, 1H), 4.03 (dd, *J* = 12.5, 5.1 Hz, 1H), 3.78 – 3.67 (m, 8H), 1.45 (s, 9H). ESI-MS: m/z 667.40 $[M+H]^+$. $C_{39}H_{42}N_2O_8$ [666.77].

***1-(3,4,5-tris(benzyloxy)benzoyl)-4-Boc piperazine-3-carboxylic acid methylate (7):***

White solid, 75% yield, melting point 109-112°C. $^1$H NMR (400 MHz, DMSO-$d_6$) δ 7.42 (dd, *J* = 7.1, 1.2 Hz, 6H), 7.35 (t, *J* = 6.7 Hz, 6H), 7.32 – 7.25 (m, 3H), 6.94 (s, 2H), 5.14 – 5.05 (m, 6H), 4.73 (t, *J* = 5.1 Hz, 1H), 4.00 (dd, *J* = 12.5, 5.1 Hz, 1H), 3.86 – 3.66 (m, 8H), 1.45 (s, 9H). ESI-MS: m/z 667.40 $[M+H]^+$. $C_{39}H_{42}N_2O_8$ [666.77]

**The general procedure for intermediates 3 and 8**:

A 250 mL round-bottom flask was charged with **2** or **8** (1.23 g, 2.51 mmol), and dissolved in 70 mL dichloromethane (DCM). Under ice bath, a solution of trifluoracetic acid (2.1 mL, 30 mmol) in 30 mL DCM was added into the system drop by drop. Stir the reaction under room temperature for 8 h, and then wash with saturated aqueous sodium hydrogen carbonate (3×60 mL) and saturated brine to remove acid. The organic

phase was dried, filtered, and concentrated in vacuo to produce the intermediate **3** or **8** as a yellow solid.

***1-(3,4,5-tris(benzyloxy)benzoyl) piperazine-2-carboxylic acid methylate (3)***

Yellow solid, 81% yield, melting point 156-158 °C. $^1$H NMR (400 MHz, DMSO-*d*$_6$) δ 7.42 (dq, J = 6.9, 1.1 Hz, 6H), 7.39 – 7.32 (m, 6H), 7.32 – 7.25 (m, 3H), 6.95 (s, 2H), 5.14 – 5.05 (m, 6H), 4.54 (t, J = 4.3 Hz, 1H), 3.83 – 3.78 (m, 1H), 3.74 – 3.69 (m, 1H), 3.68 (s, 3H), 3.16 – 3.12 (m, 1H), 3.08, 3.00 – 2.95 (m, 2H), 3.00, 2.94 – 2.83 (m, 2H). ESI-MS: m/z 565.13 $(M-H)^-$. $C_{34}H_{34}N_2O_6$ [566.77].

***1-(3,4,5-tris(benzyloxy)benzoyl) piperazine-3-carboxylic acid methylate (8)***:

Yellow solid, 80% yield, melting point 78-81°C. $^1$H NMR (400 MHz, DMSO-*d*$_6$) δ 7.42 (dq, J = 7.0, 1.1 Hz, 6H), 7.39 – 7.25 (m, 9H), 6.94 (s, 2H), 5.14 – 5.05 (m, 6H), 4.07 (dt, J = 7.3, 3.6 Hz, 1H), 3.83 (dd, J = 12.4, 3.6 Hz, 1H), 3.75 – 3.57 (m, 6H), 3.06 – 2.92 (m, 2H), 2.75 (dt, J = 7.1, 3.5 Hz, 1H). ESI-MS: m/z 565.04 $[M-1]^-$, 567.10 $[M+H]^+$. $C_{34}H_{34}N_2O_6$ [566.77].

**The general procedure for intermediates 4a, 4b and 9**:

A mixture of compound **4** or **10** (1.0 eq) and *N,N*-diisopropanyl ethylamine (DIPEA, 2.0 eq) was dissolved in 60 mL DCM, transferred to a 250 mL flask, cooled in ice bath, and sulfonyl chloride (1.5 eq) accompanied with 40 mL of DCM were added to the mixture dropwise with a constant pressure dropping funnel. The reaction was carried out under room temperature for 2 h. 50 mL saturated aqueous sodium hydrogen carbonate was used to quench the reaction when it was completed. The organic phase was separated and washed with 1 M HCl and saturated brine successively, dried over anhydrous sodium sulfate and concentrated in vacuo to acquire the intermediates **4a**, **4b** or **9** for the next step.

***1-(3,4,5-tris(benzyloxy)benzoyl)-4-(nitrobenzenesulfonyl)piperazine-2-carboxylic acid methylate (4a)***

Light-yellow solid, 77% yield, melting point 78-81°C. $^1$H NMR (400 MHz, DMSO-*d*$_6$) δ 8.35 – 8.28 (m, 2H), 7.94 – 7.86 (m, 2H), 7.42 (dq, *J* = 6.8, 1.1 Hz, 6H), 7.39 – 7.25 (m, 9H), 6.95 (s, 2H), 5.14 – 5.09 (m, 6H), 4.54 (t, *J* = 5.6 Hz, 1H), 3.91 (dd, *J* = 12.5,

6.5 Hz, 1H), 3.82 – 3.76 (m, 2H), 3.67 (dd, *J* = 12.4, 5.6 Hz, 4H), 3.29 – 3.21 (m, 2H). ESI-MS: m/z 750.09 $[M-1]^-$. $C_{40}H_{37}N_3O_{10}S$ [751.81].

***1-(3,4,5-tris(benzyloxy)benzoyl)-4-(cyanobenzenesulfonyl)piperazine-2-carboxylic acid methylate (4b)***

White solid, 81% yield, melting point 86-89°C.

***1-(3,4,5-tris(benzyloxy)benzoyl)-4-(nitrobenzenesulfonyl)piperazine-3-carboxylic acid methylate (9)***

Colorless oil, 62% yield. $^1$H NMR (400 MHz, DMSO-*d6*) δ 8.44 (d, *J* = 8.8 Hz, 2H), 8.08 (d, *J* = 8.9 Hz, 2H), 7.49 – 7.25 (m, 15H), 6.70 (s, 2H), 5.13 (s, 4H), 5.00 (s, 2H), 4.02 (d, *J* = 7.1 Hz, 1H), 3.71 (s, 3H), 3.15 (s, 2H), 2.92 (d, *J* = 20.8 Hz, 2H), 1.18 (t, *J* = 7.1 Hz, 2H). ESI-MS: m/z 750.91 $[M+H]^+$. $C_{40}H_{37}N_3O_{10}S$ [751.22].

**The general procedure for intermediates 5a, 5b and 10**:

Compound **5** or **11** (1.0 eq) was dissolved by 20 mL tetrahydrofuran (THF) and 20 mL water in a 250 mL flask, cooled to 0°C under ice bath. Then, LiOH (4.0 eq) was added and dissolved in mixture altogether. After 2 h, organic solvent was removed under reduced pressure, and add 1M HCl dropwise under stirring until pH = 4-5. Filter the solution to afford a light-yellow precipitate, which was washed by water and dried successively for next step.

***1-(3,4,5-tris(benzyloxy)benzoyl)-4-nitrosulfonyl piperazine-2-carboxylic acid (5a)***

Light-yellow solid, 96% yield, Melting point 123.5-126.5°C. $^1$H NMR (400 MHz, DMSO-*d6*) δ 8.35 (d, *J* = 8.8 Hz, 2H), 7.92 (d, *J* = 8.9 Hz, 2H), 7.42 (dq, J = 6.8, 1.1 Hz, 6H), 7.39 – 7.25 (m, 9H), 6.95 (s, 2H), 5.14 – 5.05 (m, 6H), 4.54 (t, J = 5.6 Hz, 1H), 3.91 (ddd, J = 12.5, 6.5, 4.7 Hz, 1H), 3.82 – 3.79 (m, 2H), 3.67 (dd, J = 12.4, 5.6 Hz, 1H), 3.40 – 3.31 (m, 2H). ESI-MS: m/z 736.19 $[M-1]^-$. $C_{39}H_{35}N_3O_{10}S$ [737.20].

***1-(3,4,5-tris(benzyloxy)benzoyl)-4-cyanosulfonyl piperazine-2-carboxylic acid (5b)***

Offwhite solid, 94% yield, Melting point 129-131 °C.

***1-(3,4,5-tris(benzyloxy)benzoyl)-4-nitrosulfonyl piperazine-3-carboxylic acid (10)***

Light-yellow solid, 94% yield, melting point 115-118°C. $^1$H NMR (400 MHz, DMSO-*d6*) δ 8.35 (d, *J* = 8.8 Hz, 2H), 8.06 (d, *J* = 8.9 Hz, 2H), 7.42 (dq, J = 6.8, 1.1 Hz, 6H), 7.39 – 7.25 (m, 9H), 6.94 (s, 2H), 5.14 – 5.05 (m, 6H), 4.42 (dd, J = 12.3, 6.0 Hz, 2H),

3.62 – 3.43 (m, 3H), 2.89 – 2.79 (m, 2H). ESI-MS: m/z 736.13 [M-1]$^-$. $C_{39}H_{35}N_3O_{10}S$ [737.78].

**The general procedure for intermediates 6a, 6b and 11**:

Compound **6** or **12** (1.0 eq) was suspended in DCM together with HATU (1.5 eq) under ice bath. Then, DIPEA (3.0 eq) and various amines (1.5 eq) were added in the mixture, and reacted under room temperature for 8 h. When the reaction was finished, the organic phase was washed with 1M HCl, saturated aqueous sodium hydrogen carbonate solution, and saturated brine successively. The organic phase was dried over anhydrous sodium sulfate, concentrated, and purified by flash chromatography to produce **6a**, **6b** and **11** series of compounds.

***N-phenyl-4-((4-nitrophenyl)sulfonyl)-1-(3,4,5-tris(benzyloxy)benzoyl)piperazine-2-carboxamide*** (from series **6a**).

White solid, 73% yield, melting point 105-108°C. $^1$H NMR (400 MHz, DMSO-$d_6$) δ 9.33 (s, 1H), 8.32 (d, $J$ = 8.8 Hz, 2H), 7.92 (d, $J$ = 8.9 Hz, 2H), 7.54 – 7.49 (m, 2H), 7.42 (dq, $J$ = 7.1, 1.1 Hz, 6H), 7.35 (tt, $J$ = 7.1, 1.3 Hz, 8H), 7.32 – 7.26 (m, 3H), 7.08 (tt, $J$ = 7.0, 1.2 Hz, 1H), 6.97 (s, 2H), 5.14 – 5.05 (m, 6H), 4.91 (t, $J$ = 5.0 Hz, 1H), 3.95 (ddd, $J$ = 12.5, 6.7, 4.6 Hz, 1H), 3.87 – 3.79 (m, 2H), 3.68 (dd, $J$ = 12.4, 5.0 Hz, 1H), 3.29 – 3.21 (m, 2H). ESI-MS: m/z 812.14 [M-1]$^-$. $C_{45}H_{40}N_4O_9S$ [813.25].

**N-phenyl-4-((4-cyanophenyl)sulfonyl)-1-(3,4,5-tris(benzyloxy)benzoyl)piperazine-2-carboxamide (6b)**.

White solid, 71 % yield, melting point 112-114 °C.

***N-phenyl-4-((4-nitrophenyl)sulfonyl)-1-(3,4,5-tris(benzyloxy)benzoyl)piperazine-2-carboxamide*** *(*from series **11***)*.

White solid, 84% yield, melting point 153-156°C. $^1$H NMR (400 MHz, DMSO-$d_6$) δ 9.43 (s, 1H), 8.35 (d, $J$ = 8.8 Hz, 2H), 8.06 (d, $J$ = 8.9 Hz, 2H), 7.55 – 7.49 (m, 2H), 7.42 (dq, $J$ = 7.1, 1.1 Hz, 6H), 7.35 (tt, $J$ = 7.1, 1.2 Hz, 8H), 7.32 – 7.26 (m, 3H), 7.08 (tt, $J$ = 7.0, 1.2 Hz, 1H), 6.94 (s, 2H), 5.14 – 5.05 (m, 6H), 4.61 (t, $J$ = 5.4 Hz, 1H), 3.89 – 3.77 (m, 3H), 3.64 – 3.58 (m, 2H), 3.56 – 3.48 (m, 1H). ESI-MS: m/z 812.14 [M-1]$^-$. $C_{45}H_{40}N_4O_9S$ [813.25].

**The general procedure for compounds DF-35, DF-36, DF-47, DF-57, DF-63, DF-**

**64, DF-67, DF-68, DF-69, DF-71).**

Dissolve compound **6a** or **11** in 10 mL methanol, and add 10% (w/w) Pd/C catalyst (55% water) to the system. Stir the suspension under hydrogen atmosphere for 20 h, then filtered through a celite pad, and concentrated in vacuo. Crude products were recrystallized from EtOAc to yield final product DF-35, DF-36, DF-47, DF-57, DF-63, DF-64, DF-67, DF-69, DF-71, DF-75.

***N-(4-methoxybenzyl)-1-(3,4,5-trihydroxybenzonyl)-4-(4-amniobenzylsulfonyl) piperazine-2-carboxamide (DF-35)***

White solid, 86% yield, purity 98%, melting point 192-195 °C. $^{1}$H NMR (400 MHz, DMSO-$d_6$) δ 9.11 (s, 2H), 8.51 (t, *J* = 5.8 Hz, 2H), 7.56 (t, *J* = 5.8 Hz, 2H), 7.53 – 7.48 (m, 2H), 7.42 (s, 2H), 7.17 (dt, *J* = 8.5, 1.0 Hz, 2H), 6.88 – 6.82 (m, 2H), 6.78 (s, 1H), 4.27 (t, *J* = 5.2 Hz, 3H), 4.32 (d, *J* = 5.7 Hz, 1H), 3.60 (s, 3H), 3.40 – 3.29 (m, 2H), 2.73 (s, 1H), 2.32 (s, 1H), 2.10 (s, 1H). $^{13}$C NMR (100 MHz, DMSO-$d_6$) δ 170.44, 169.87, 158.96, 152.95, 146.19, 138.75, 134.12, 131.67, 129.16, 129.08, 128.36, 114.30, 113.92, 106.67, 55.32, 52.88, 48.62, 45.87, 45.82, 43.71. ESI-MS: m/z 555.34 $[M-1]^-$. $C_{26}H_{28}N_4O_8S$ [556.59].

***N-(furan-2-ylmethyl)-1-(3,4,5-trihydroxybenzonyl)-4-(4-amniobenzylsulfonyl) piperazine-2-carboxamide (DF-36).***

White solid, 82% yield, purity 97.5%, melting point 181-184 °C. $^{1}$H NMR (400 MHz, DMSO-$d_6$) δ 9.08 (s, 2H), 8.48 (s, 1H), 7.66 (t, *J* = 4.9 Hz, 1H), 7.54 – 7.48 (m, 2H), 7.42 (s, 2H), 7.33 (t, *J* = 1.6 Hz, 2H), 6.78 (s, 2H), 6.67 – 6.61 (m, 2H), 4.43 – 4.36 (m, 1H), 4.36 – 4.28 (m, 3H), 3.92 (ddd, *J* = 12.3, 6.6, 4.7 Hz, 1H), 3.77 (dd, *J* = 12.3, 4.6 Hz, 1H), 3.71 (dd, *J* = 12.4, 5.2 Hz, 1H), 3.60 (dd, *J* = 12.4, 5.2 Hz, 1H), 3.40 – 3.29 (m, 2H). $^{13}$C NMR (100 MHz, DMSO-$d_6$) δ 170.45, 169.87, 153.82, 152.95, 146.19, 142.29, 138.75, 131.67, 129.08, 128.36, 114.30, 110.56, 107.58, 106.67, 52.88, 48.61, 45.87, 28.98. ESI-MS: m/z 515.23 $[M-1]^-$. $C_{23}H_{24}N_4O_8S$ [516.53].

***N-([1,1'-biphenyl]-4-ylmethyl)-1-(3,4,5-trihydroxybenzonyl)-4-(4-amniobenzyl sulfonyl) piperazine-2-carboxamide (DF-47).***

White solid, 62% yield, purity 97%, melting point 193-196 °C. $^{1}$H NMR (400 MHz,

DMSO-$d_6$) δ 9.12 (s, 2H), 8.60 (t, $J$ = 5.9 Hz, 1H), 8.52 (s, 1H), 7.68 – 7.62 (m, 3H), 7.51 – 7.31 (m, 8H), 6.67 (d, $J$ = 8.7 Hz, 2H), 6.31 (s, 2H), 6.15 (s, 2H), 4.40 (s, 2H), 4.11 (d, $J$ = 7.7 Hz, 1H), 3.51 (s, 3H), 2.37 (d, $J$ = 32.4 Hz, 1H), 2.20 – 2.07 (m, 1H), 1.99 (s, 1H). $^{13}$C NMR (100 MHz, DMSO-$d_6$) δ 171.33, 168.89, 153.91, 146.19, 140.48, 139.12, 135.31, 130.11, 129.40, 128.06, 127.77, 127.08, 127.05, 119.31, 113.28, 106.67, 52.88, 48.62, 45.87, 42.48. ESI-MS: m/z 603.74 [M+H]$^+$. $C_{31}H_{30}N_4O_7S$ [602.68].

***N-phenyl-1-(3,4,5-trihydroxybenzonyl)-4-(4-cyanobenzylsulfonyl)piperazine-3-carboxamide (DF-57).***

White solid, 75% yield, purity 98.2%, melting point 173-176 °C. $^1$H NMR (400 MHz, DMSO-$d_6$) δ 10.03 (s, 1H), 9.07 (s, 2H), 8.43 (s, 1H), 7.37 (d, $J$ = 8.4 Hz, 4H), 7.28 (t, $J$ = 7.8 Hz, 2H), 7.05 (t, $J$ = 7.3 Hz, 1H), 6.53 (d, $J$ = 8.5 Hz, 2H), 6.24 (s, 2H), 6.02 (s, 2H, $NH_2$), 4.49 (s, 1H), 3.78 (d, $J$ = 11.5 Hz, 2H), 3.46 (d, $J$ = 9.2 Hz, 1H), 3.15 (d, $J$ = 19.4 Hz, 2H), 3.05 (d, $J$ = 11.9 Hz, 1H). $^{13}$C NMR (100 MHz, DMSO-$d_6$) δ 170.16, 168.03, 153.52, 146.06, 138.75, 135.13, 129.41, 129.07, 125.50, 124.02, 123.67, 120.37, 113.14, 106.74, 54.87, 42.26, 34.85. ESI-MS: m/z 511.22 [M-1]$^-$. $C_{24}H_{24}N_4O_7S$ [512.54].

***N-methyl-N-phenyl-1-(3,4,5-trihydroxybenzonyl)-4-(4-cyanobenzylsulfonyl) piperazine-3-carboxamide (DF-63).***

White solid, 61% yield, purity 95.6%, melting point 172-175 °C. $^1$H NMR (400 MHz, DMSO-$d_6$) δ 9.23 (s, 2H), 9.02 (s, 1H), 7.61 (d, $J$ = 8.5 Hz, 2H), 7.38 – 7.32 (m, 5H), 6.89 (d, $J$ = 8.7 Hz, 2H), 6.62 (d, $J$ = 8.1 Hz, 2H), 6.09 (s, 2H, $NH_2$), 5.12 (s, 3H), 4.97 – 4.89 (m, 1H), 4.04 (t, $J$ = 5.7 Hz, 2H), 3.01 (s, 4H). $^{13}$C NMR (100 MHz, DMSO-$d_6$) δ 169.77, 169.45, 152.90, 146.20, 142.18, 138.75, 131.13, 129.16, 128.89, 128.35, 126.45, 123.98, 114.23, 106.89, 55.57, 46.55, 45.49, 45.10, 32.79. ESI-MS: m/z 525.57 [M-1]$^-$. $C_{25}H_{26}N_4O_7S$ [526.56].

***N-(4-methoxybenzyl)-1-(3,4,5-trihydroxybenzonyl)-4-(4-amniobenzylsulfonyl) piperazine-3-carboxamide (DF-64)***

White solid, 69% yield, purity 95.9%, melting point 202-205 °C. $^1$H NMR (400 MHz, DMSO-$d_6$) δ 9.08 (s, 2H), 8.49 (s, 1H), 8.28 (s, 1H), 7.37 (d, $J$ = 8.4 Hz, 2H), 7.05 (d,

$J$ = 7.1 Hz, 2H), 6.86 (d, $J$ = 8.3 Hz, 2H), 6.59 (d, $J$ = 8.5 Hz, 2H), 6.25 (s, 2H), 6.04 (s, 2H, $NH_2$), 4.30 (s, 2H), 4.09 (t, $J$ = 7.2 Hz, 1H), 4.06 – 3.85 (m, 2H), 3.73 (s, 3H), 3.49 (d, $J$ = 12.7 Hz, 2H), 2.99 – 2.76 (m, 2H). $^{13}$C NMR (100 MHz, DMSO-$d_6$) δ 170.26, 169.00, 158.72, 153.50, 146.01, 135.20, 131.22, 129.48, 129.11, 125.62, 124.00, 114.16, 113.14, 106.95, 55.53, 54.68, 42.07, 40.45. ESI-MS: m/z 555.79 [M-1]$^-$. $C_{26}H_{28}N_4O_8S$ [556.59].

***N-(naphthalen-1-ylmethyl)-1-(3,4,5-trihydroxybenzonyl)-4-(4-amniobenzyl sulfonyl) piperazine-3-carboxamide (DF-67)***

White solid, 67% yield, purity 97.8%, melting point 189-192 °C. $^1$H NMR (400 MHz, DMSO-$d_6$) δ 9.15 (s, 2H), 8.54 (s, 2H), 7.98 – 7.83 (m, 3H), 7.59 – 7.52 (m, 2H), 7.41 (p, $J$ = 9.2, 8.5 Hz, 4H), 6.60 (d, $J$ = 8.6 Hz, 2H), 6.29 (s, 2H), 6.06 (s, 2H, $NH_2$), 4.36 (s, 2H), 4.03 (q, $J$ = 7.1 Hz, 1H), 3.75 – 3.49 (m, 2H), 2.86 (d, $J$ = 23.8 Hz, 2H), 2.71 (d, $J$ = 16.9 Hz, 2H). $^{13}$C NMR (100 MHz, DMSO-$d_6$) δ 170.40, 169.21, 153.52, 146.06, 135.19, 134.24, 133.68, 131.34, 129.47, 128.93, 128.09, 126.84, 126.29, 125.92, 124.01, 123.86, 113.12, 106.89, 60.24, 54.59, 21.24. ESI-MS: m/z 575.86 [M-1]$^-$. $C_{29}H_{28}N_4O_8S$ [576.17].

***N-(thiophen-2-ylmethyl)-1-(3,4,5-trihydroxybenzonyl)-4-(4-amniobenzylsulfonyl) piperazine-3-carboxamide (DF-68).***

White solid, 72% yield, purity 96.4%, melting point 213-216 °C. $^1$H NMR (400 MHz, DMSO-$d_6$) δ 8.75 (s, 2H), 8.19 (s, 1H), 7.62 – 7.56 (m, 5H), 7.42 (d, $J$ = 23.8 Hz, 2H), 7.01 – 6.92 (m, 2H), 6.12 (s, 2H, $NH_2$), 4.49 (dd, $J$ = 14.7, 4.8 Hz, 2H), 4.32 (d, $J$ = 5.7 Hz, 2H), 4.03 (q, $J$ = 7.1 Hz, 1H), 4.00 (dd, $J$ = 12.5, 5.7 Hz, 1H), 3.83 (ddd, $J$ = 12.1, 7.4, 4.6 Hz, 2H), 3.75 – 3.66 (m, 2H). $^{13}$C NMR (100 MHz, DMSO-$d_6$) δ 161.03, 140.45, 128.89, 128.61, 128.54, 128.36, 128.07, 127.56, 127.27, 126.77, 74.77, 70.76, 54.08, 43.14, 42.31. ESI-MS: m/z 531.84 [M-1]$^-$. $C_{23}H_{24}N_4O_7S_2$ [532.59].

***N-([1,1'-biphenyl]-4-ylmethyl)-1-(3,4,5-trihydroxybenzonyl)-4-(4-amniobenzyl sulfonyl) piperazine-3-carboxamide (DF-69).***

White solid, 67% yield, purity 95.0%, melting point 192-195 °C. $^1$H NMR (400 MHz, DMSO-$d_6$) δ 9.13 (s, 2H), 8.65 (s, 1H), 8.57 (s, 1H), 8.16 (d, $J$ = 8.4 Hz, 2H), 7.90 (d,

*J* = 8.4 Hz, 2H), 7.64 (dd, *J* = 15.2, 7.8 Hz, 5H), 7.47 (s, 2H), 7.37 (d, *J* = 6.0 Hz, 2H), 6.33 (s, 2H, $NH_2$), 4.30 (d, *J* = 69.7 Hz, 5H), 3.67 – 3.56 (m, 3H), 3.13 (d, *J* = 6.2 Hz, 1H). $^{13}$C NMR (100 MHz, DMSO-$d_6$) δ 171.22, 146.19, 140.45, 139.16, 135.35, 134.16, 129.08, 128.74, 127.58, 127.07, 118.12, 116.26, 54.05, 48.62, 42.42. ESI-MS: m/z 601.5 $[M-1]^-$. $C_{31}H_{30}N_4O_7S$ [602.18].

***N-(7-Boc-2,7-diazaspiro[4.4]nonan-2-yl)-1-(3,4,5-trihydroxybenzonyl)-4-(4-amniobenzylsulfonyl) piperazine-3-carboxamide (DF-71).***

White solid, 67% yield, purity 96.7%, melting point 192-195 °C. $^1$H NMR (400 MHz, DMSO-$d_6$) δ 9.10 (s, 2H), 8.45 (s, 1H), 7.42 (d, *J* = 8.4 Hz, 2H), 7.31 (s, 2H), 6.67 – 6.61 (m, 2H), 6.08 (s, 2H, $NH_2$), 4.36 – 4.29 (m, 2H), 3.88 – 3.78 (m, 1H), 3.75 – 3.71 (m, 1H), 3.71 – 3.63 (m, 2H), 3.61 – 3.36 (m, 9H), 1.90 – 1.78 (m, 2H), 1.73 (dddd, *J* = 12.3, 8.0, 4.8, 3.1 Hz, 2H), 1.47 (s, 9H). $^{13}$C NMR (100 MHz, DMSO-$d_6$) δ 169.84, 169.45, 155.75, 152.90, 146.20, 138.75, 131.15, 129.16, 128.35, 114.23, 106.89, 79.75, 59.60, 59.06, 57.54, 49.16, 47.29, 47.15, 46.60, 45.49, 45.10, 37.71, 37.66, 28.39. ESI-MS: m/z 644.86 $[M-1]^-$. $C_{30}H_{39}N_5O_9S$ [645.25].

***N-([1,1'-biphenyl]-4-ylmethyl)-4-((4-cyanophenyl)sulfonyl)-1-(3,4,5-trihydroxy benzoyl)piperazine-2-carboxamide (DF-51).***

Dissolve compound **6b** (0.59 g, 0.669 mmol) in 20 mL of DCM under a -45 °C cold bath, and $BCl_3$ (0.23 g, 2.0 mmol) was added to the system dropwise with stirring. When the reaction was finished (monitored by TLC), 2 mL methanol was added to quench the reaction. Organic phase was concentrated in vacuo to acquire crude product, which was purified through flash chromatography to yield compound DF-51. White granular solid, 74% yield, purity 98.1%, melting point 197-200 °C. $^1$H NMR (400 MHz, DMSO-$d_6$) δ 9.13 (s, 2H), 8.65 (s, 1H), 8.57 (s, 1H), 8.16 (d, *J* = 8.4 Hz, 2H), 7.90 (d, *J* = 8.4 Hz, 2H), 7.64 (dd, *J* = 15.2, 7.8 Hz, 5H), 7.47 (s, 2H), 7.37 (d, *J* = 6.0 Hz, 2H), 6.33 (s, 2H), 4.30 (d, *J* = 69.7 Hz, 5H), 3.67 – 3.56 (m, 3H), 3.13 (d, *J* = 6.2 Hz, 1H). $^{13}$C NMR (100 MHz, DMSO-$d_6$) δ 171.22, 146.19, 140.45, 139.16, 135.35, 134.16, 129.08, 128.74, 127.58, 127.07, 118.12, 116.26, 54.05, 48.62, 42.42. ESI-MS: m/z 611.13 $[M-1]^-$. $C_{32}H_{28}N_4O_7S$ [612.66].

$^{1}$H NMR (600 MHz, DMSO-$d_6$) of **DF-35**

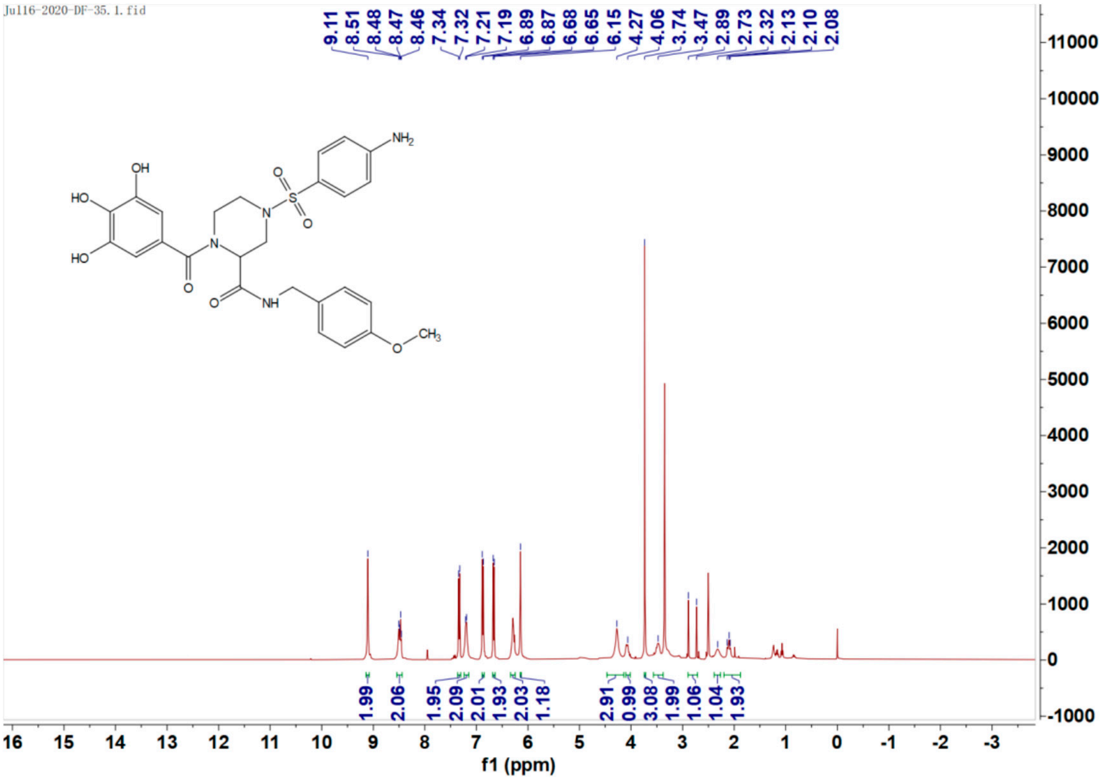

$^{13}$C NMR (150 MHz, DMSO-$d_6$) of **DF-35**

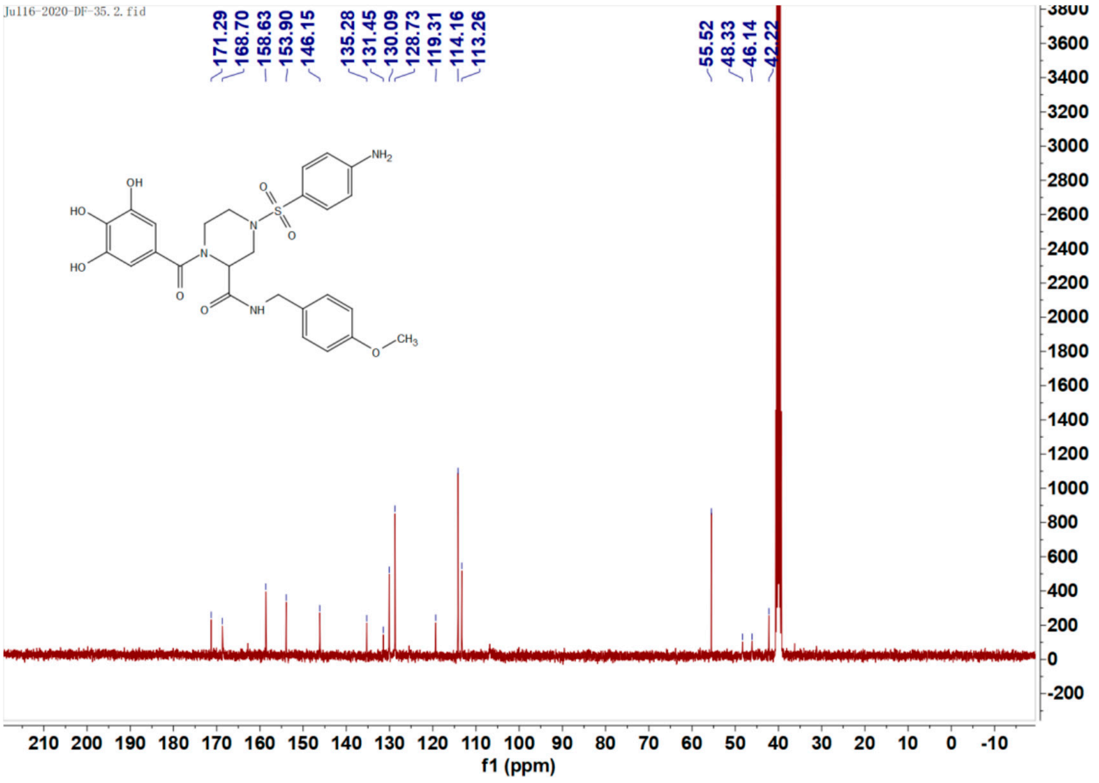

$^{1}H$ NMR (600 MHz, DMSO-$d_6$) of **DF-36**

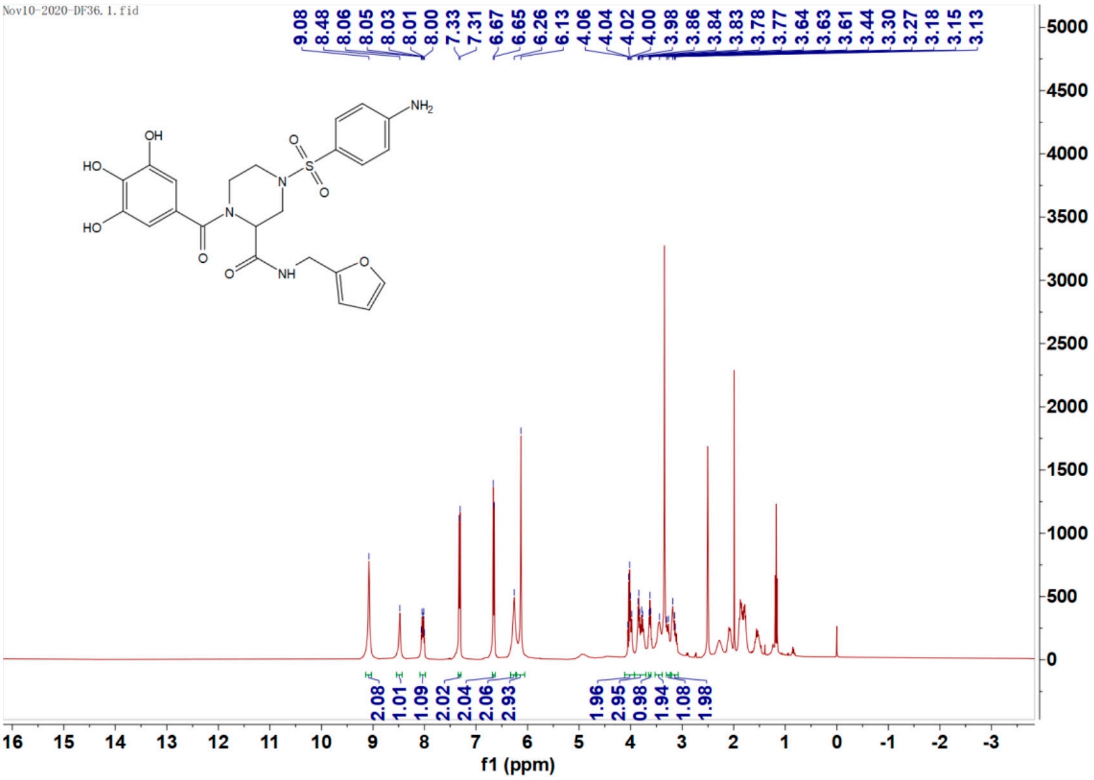


$^{13}C$ NMR (150 MHz, DMSO-$d_6$) of **DF-36**

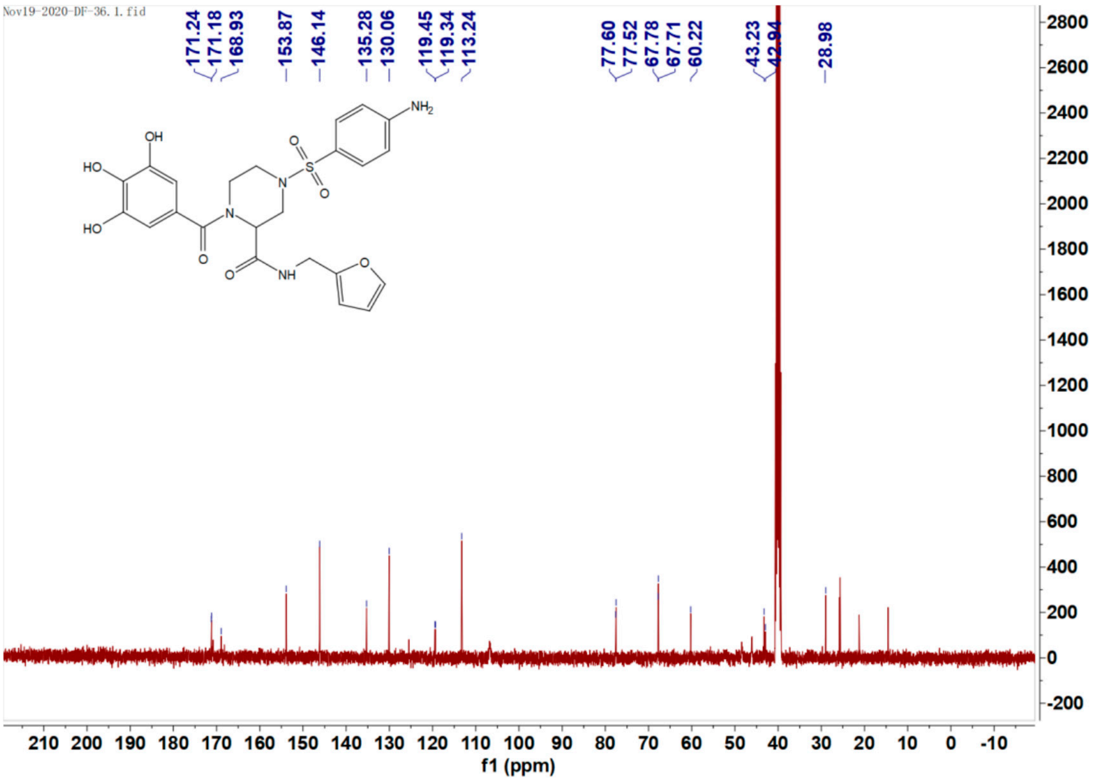

$^{1}$H NMR (600 MHz, DMSO-$d_6$) of **DF-47**

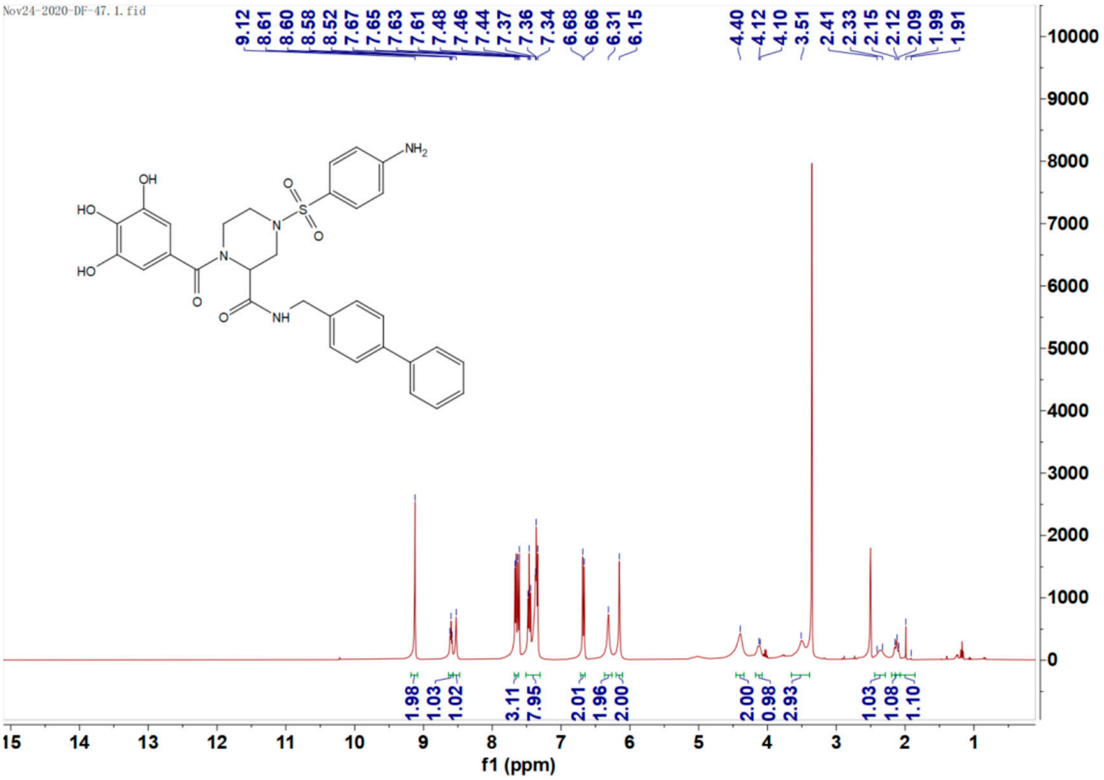


$^{13}$C NMR (150 MHz, DMSO-$d_6$) of **DF-47**

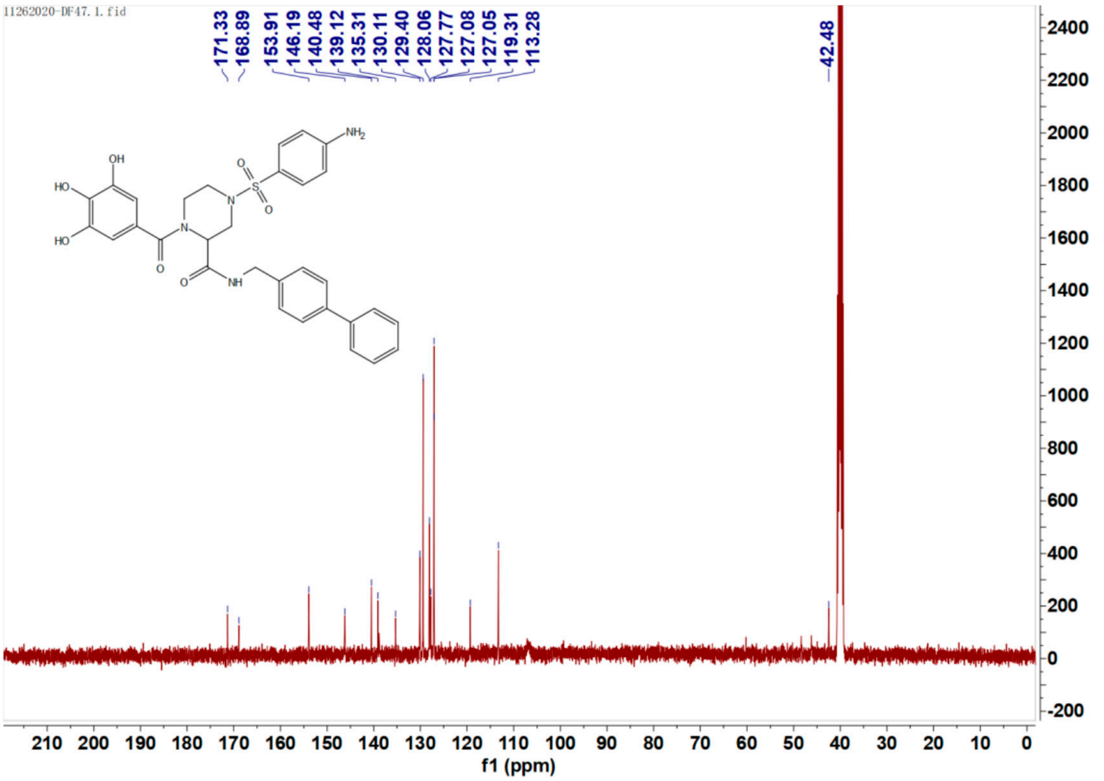

$^{1}$H NMR (600 MHz, DMSO-$d_6$) of **DF-57**

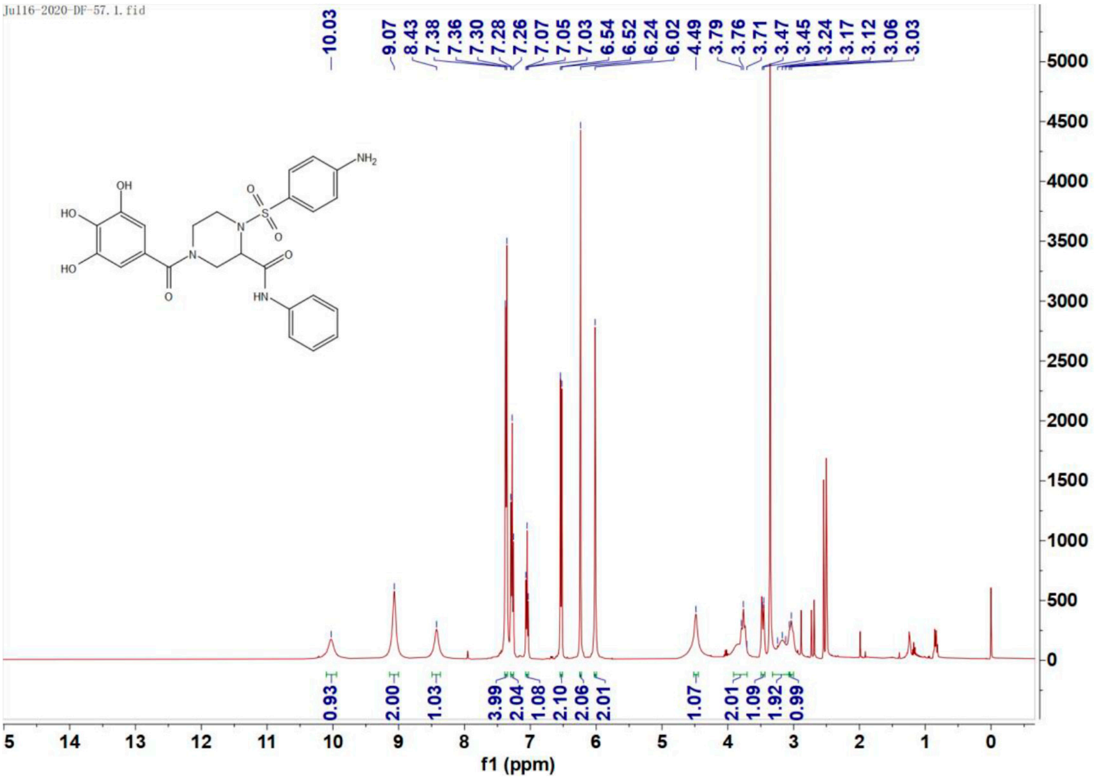


$^{13}$C NMR (150 MHz, DMSO-$d_6$) of **DF-57**

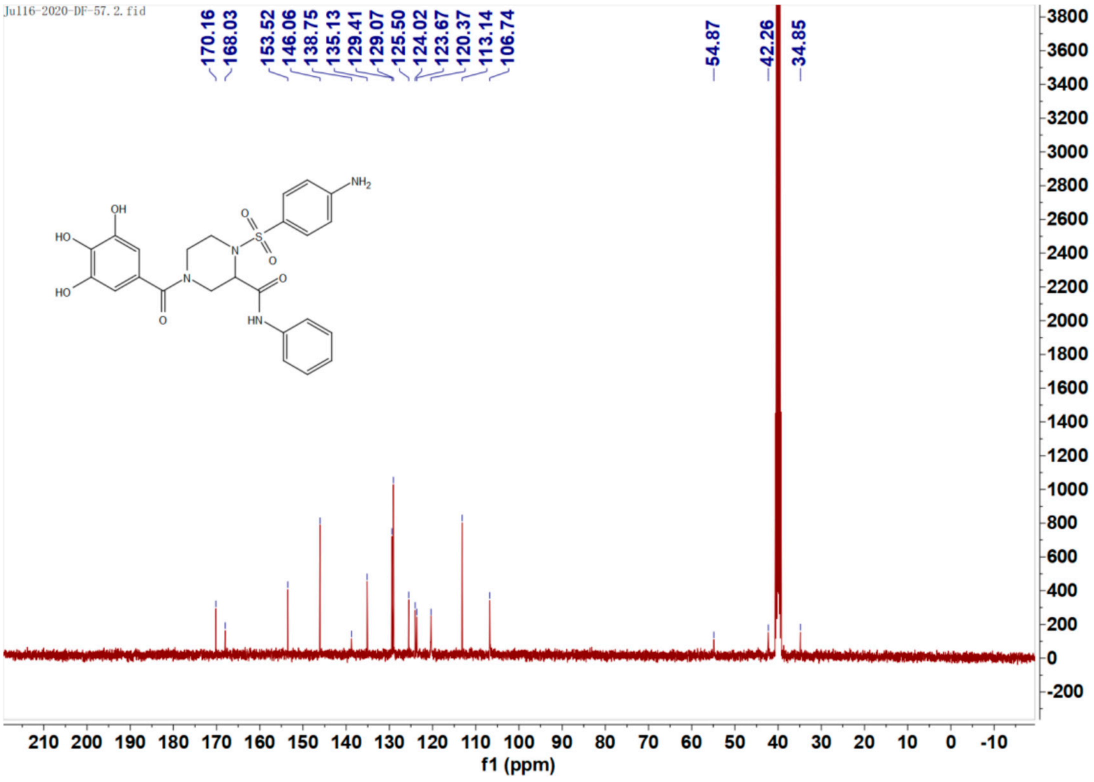

$^{1}$H NMR (600 MHz, DMSO-$d_6$) of **DF-63**

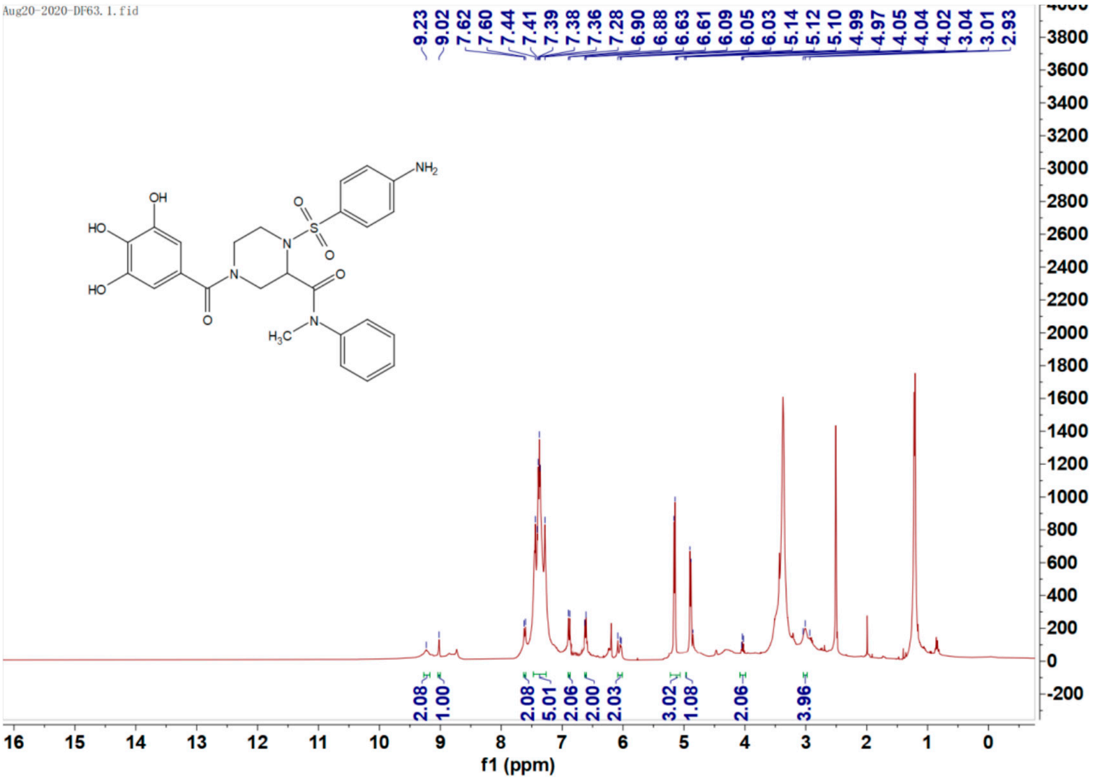


$^{13}$C NMR (150 MHz, DMSO-$d_6$) of **DF-63**

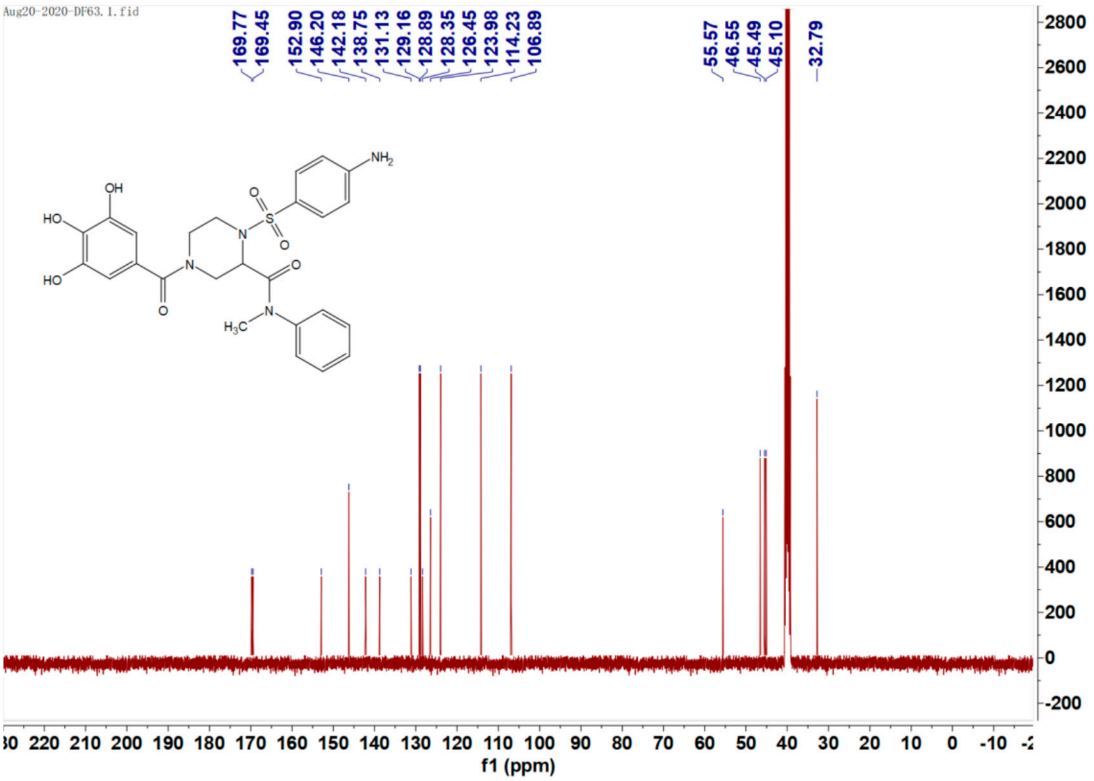

$^{1}$H NMR (600 MHz, DMSO-$d_6$) of **DF-64**

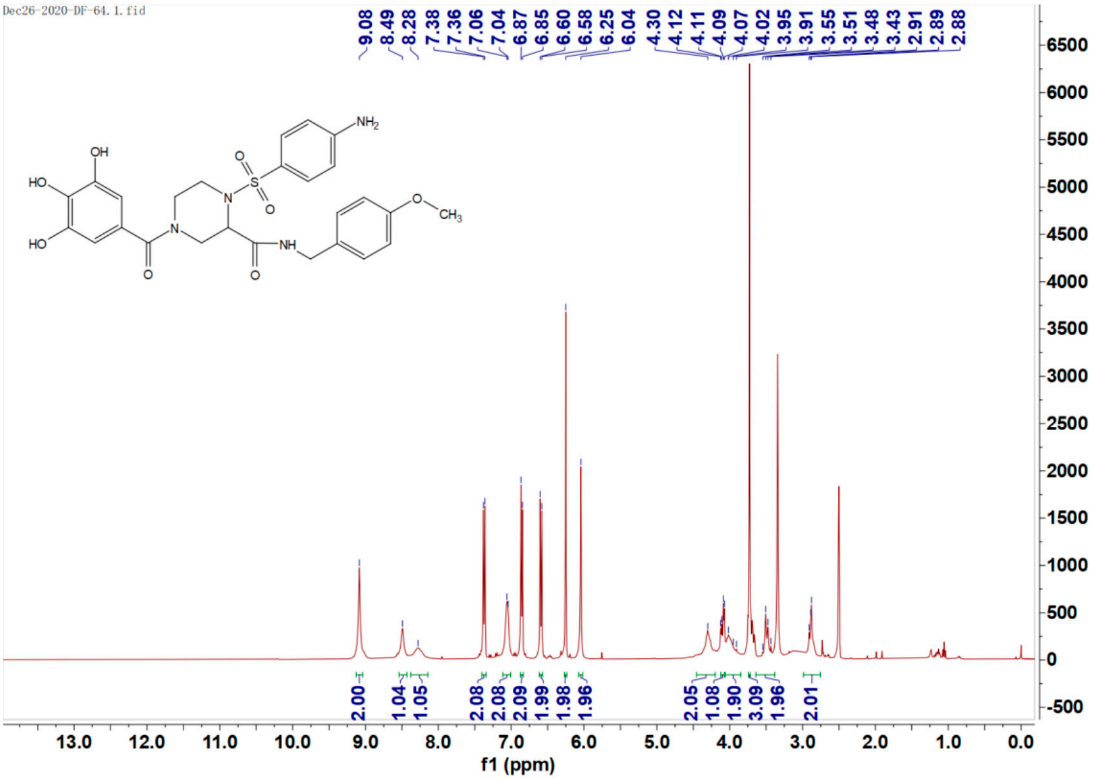

$^{13}$C NMR (150 MHz, DMSO-$d_6$) of **DF-64**

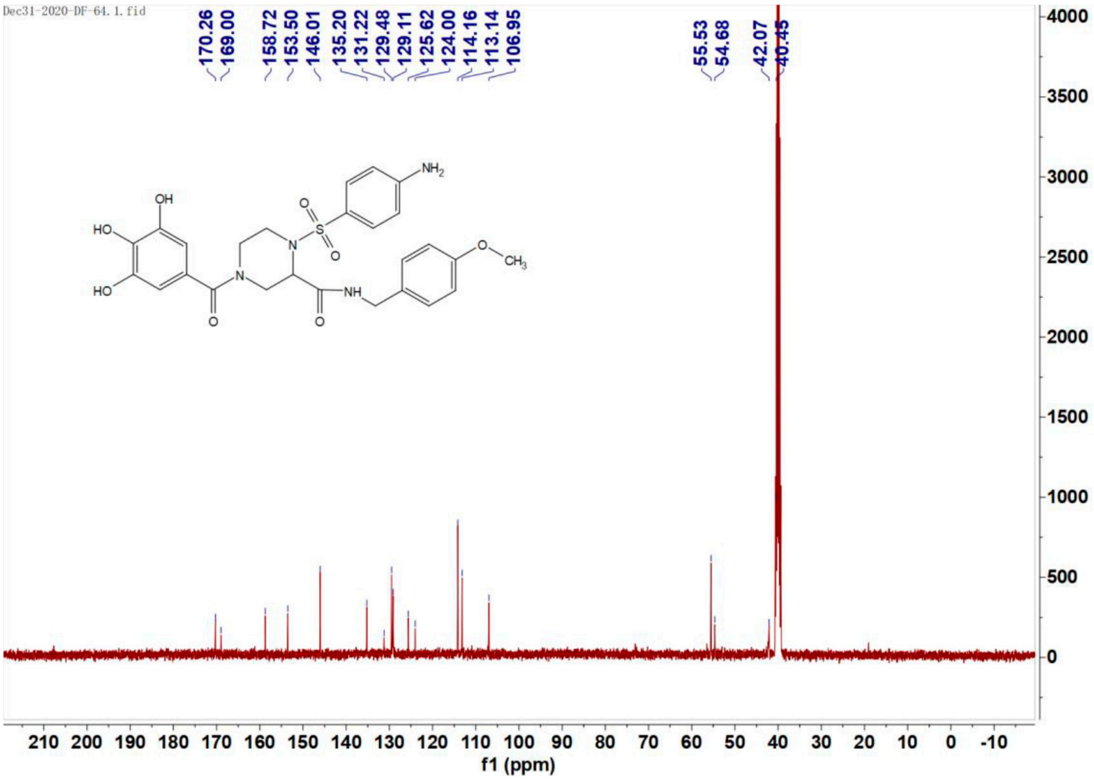

$^{1}$H NMR (600 MHz, DMSO-$d_6$) of **DF-67**

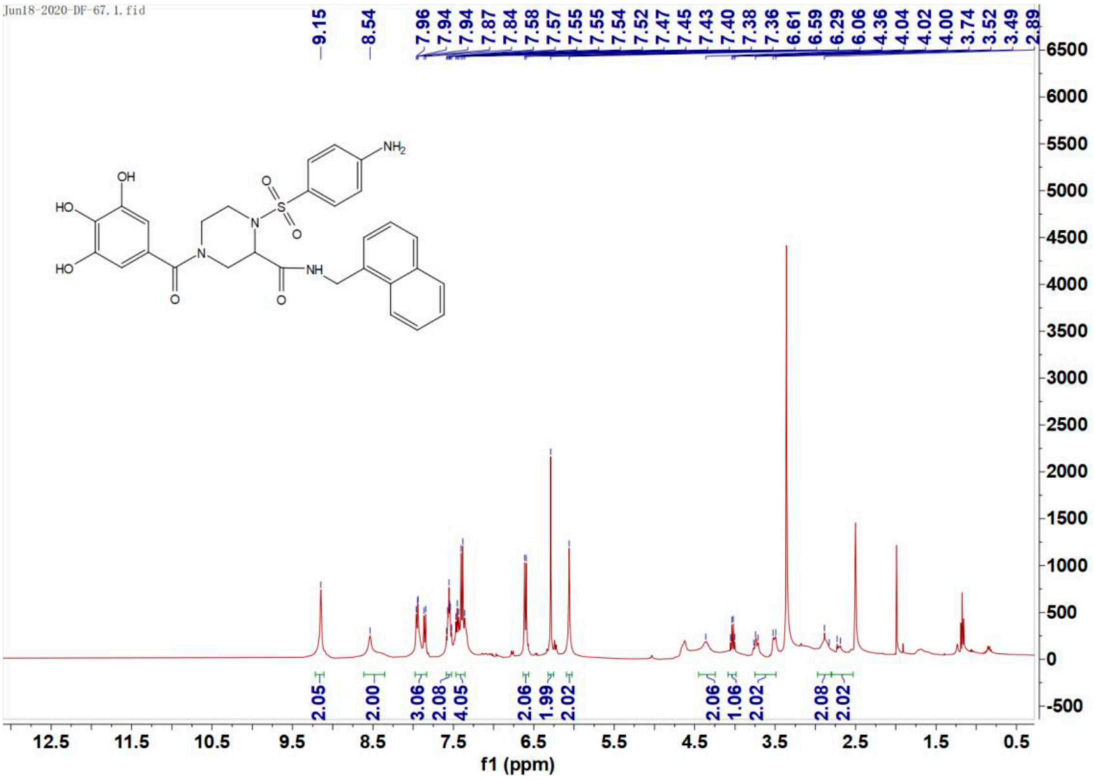

$^{13}$C NMR (150 MHz, DMSO-$d_6$) of **DF-67**

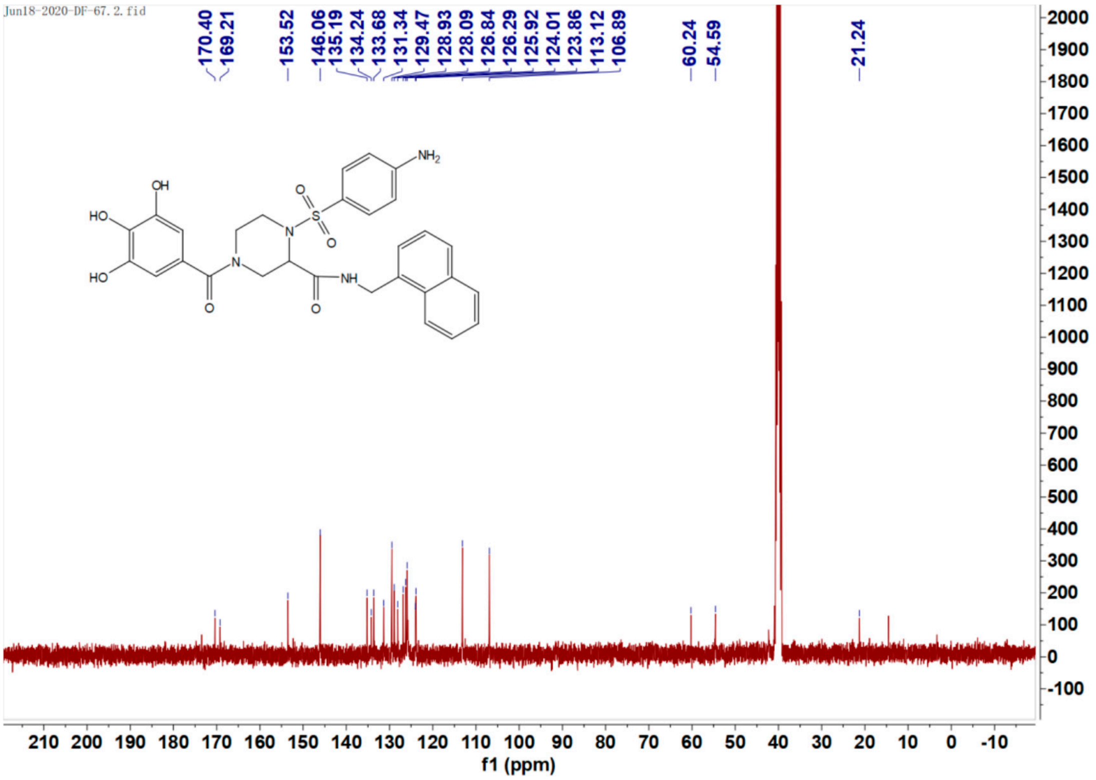

$^{1}$H NMR (600 MHz, DMSO-$d_6$) of **DF-68**

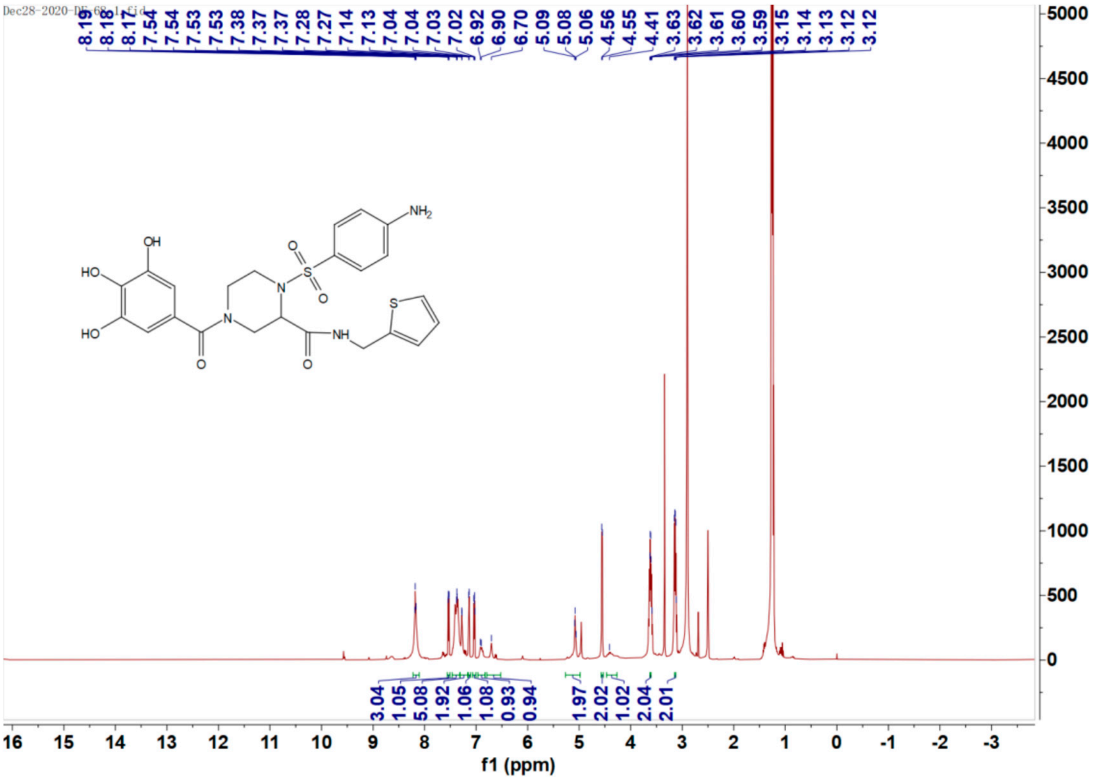


$^{13}$C NMR (150 MHz, DMSO-$d_6$) of **DF-68**

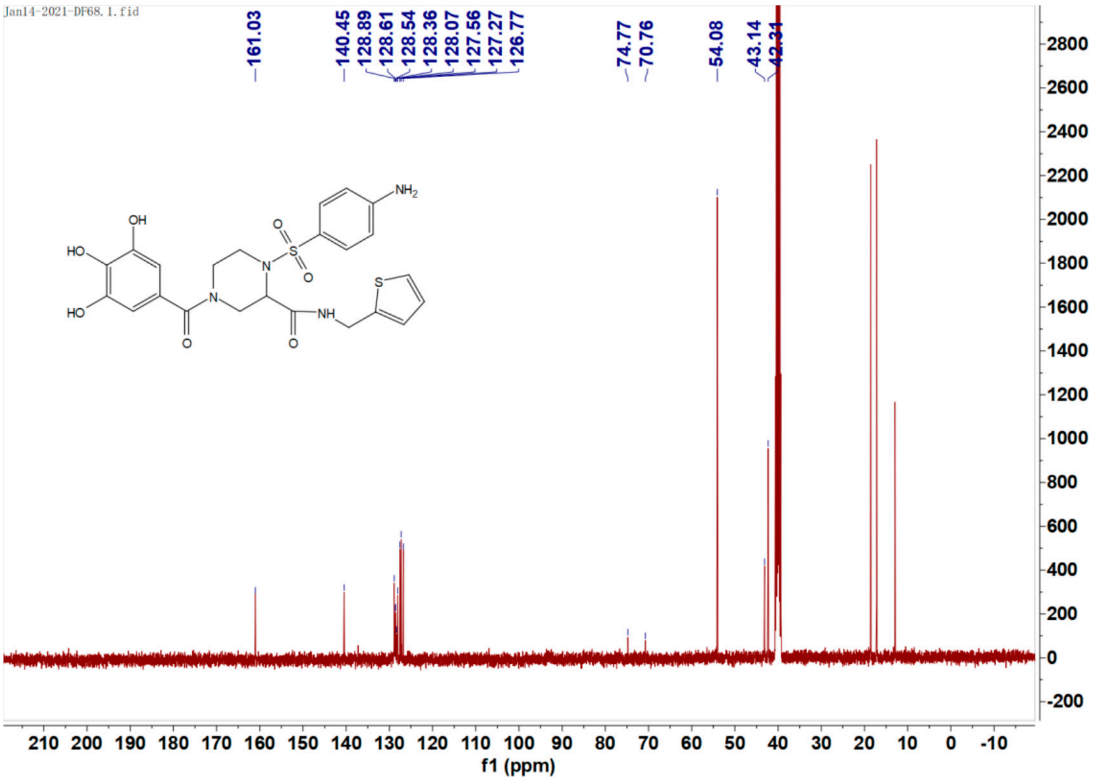

$^{1}H$ NMR (600 MHz, DMSO-$d_6$) of **DF-69**

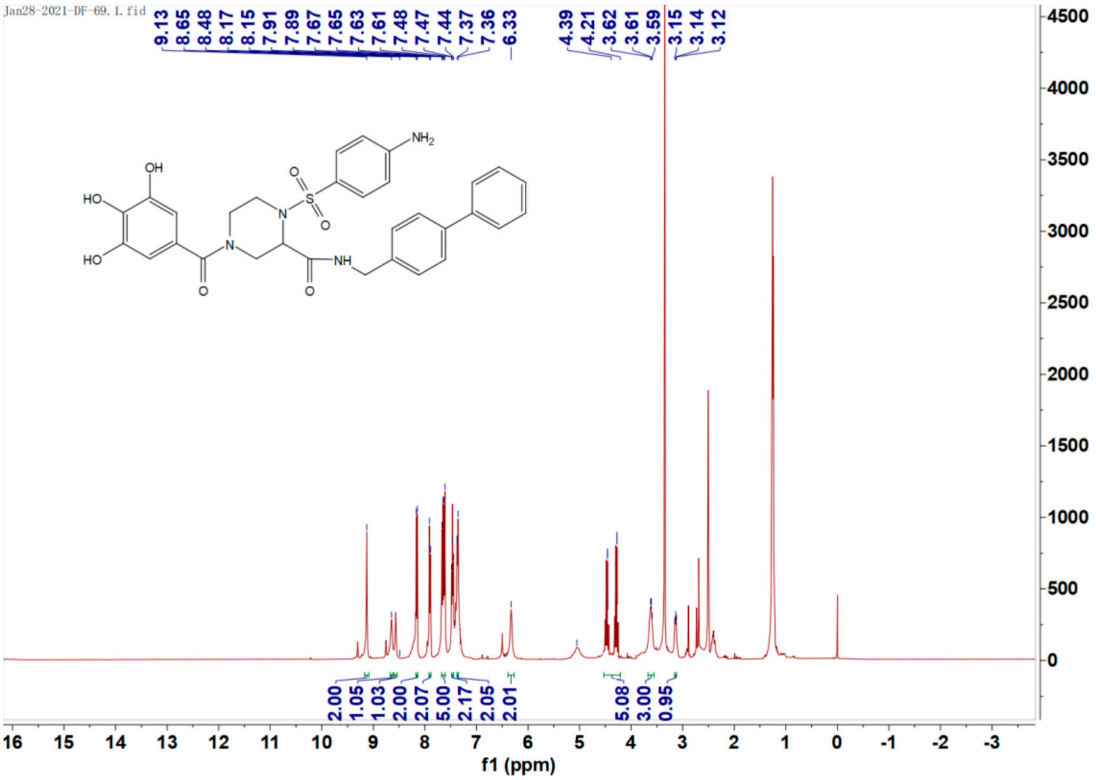

$^{13}C$ NMR (150 MHz, DMSO-$d_6$) of **DF-69**

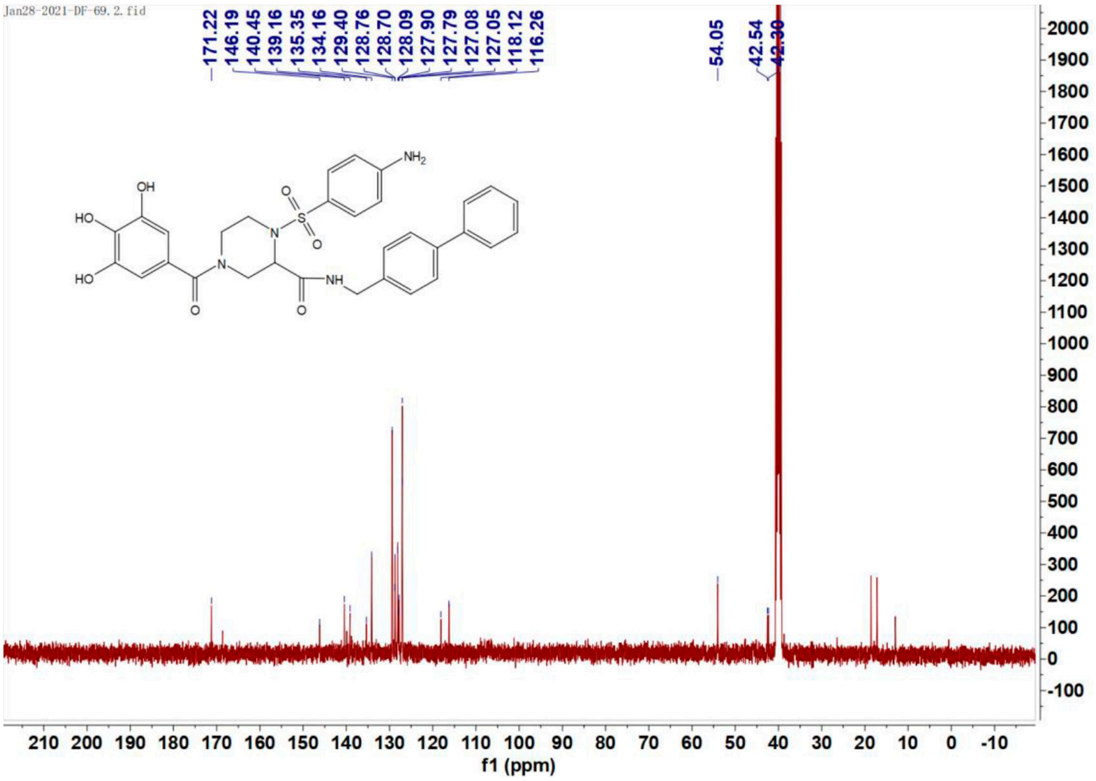

$^{1}$H NMR (600 MHz, DMSO-$d_6$) of **DF-51**

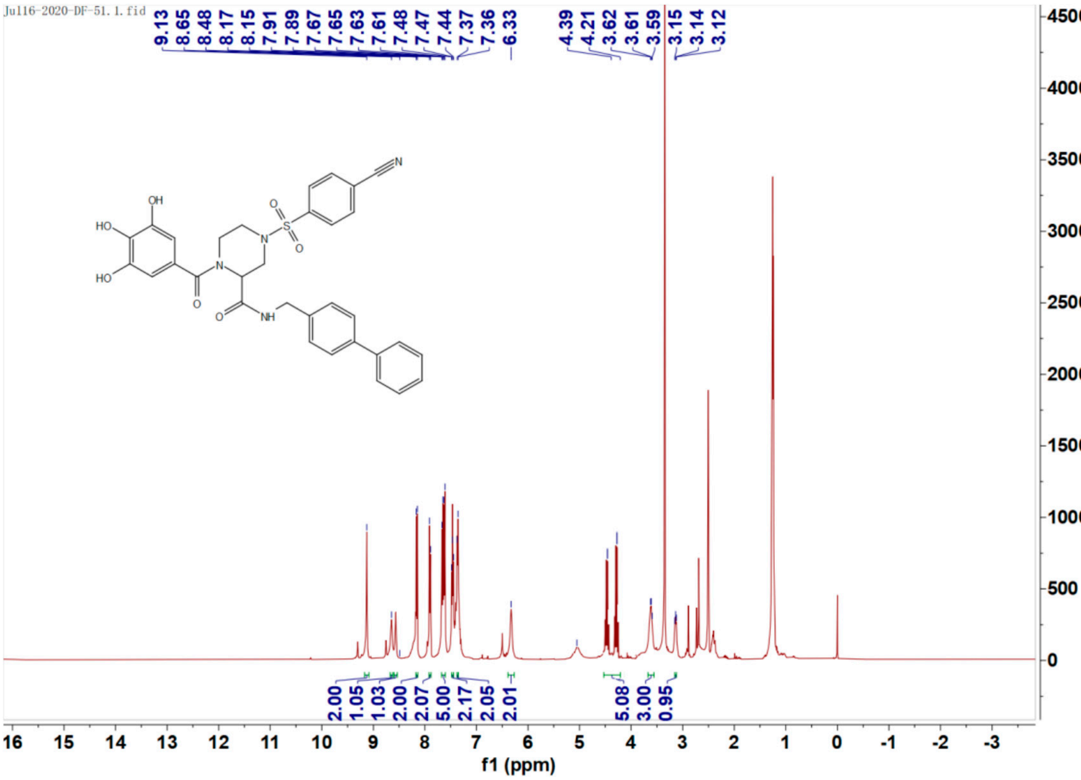


$^{13}$C NMR (150 MHz, DMSO-$d_6$) of **DF-51**

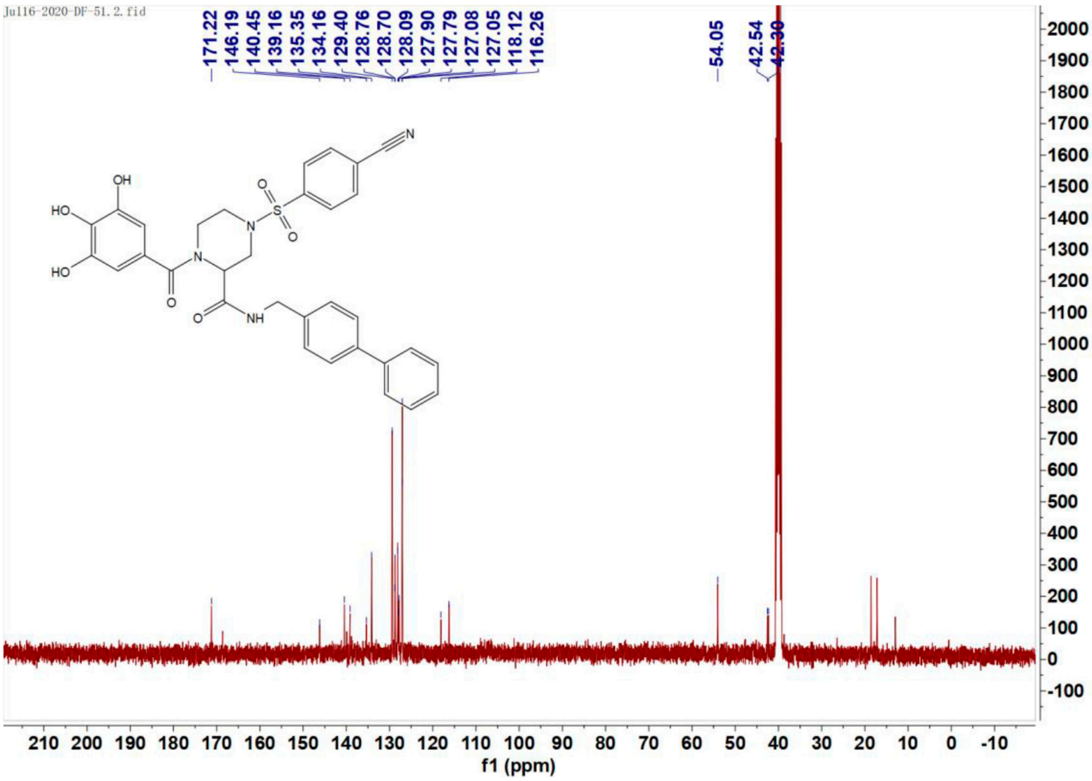

Mass spectrometry of **DF-35**

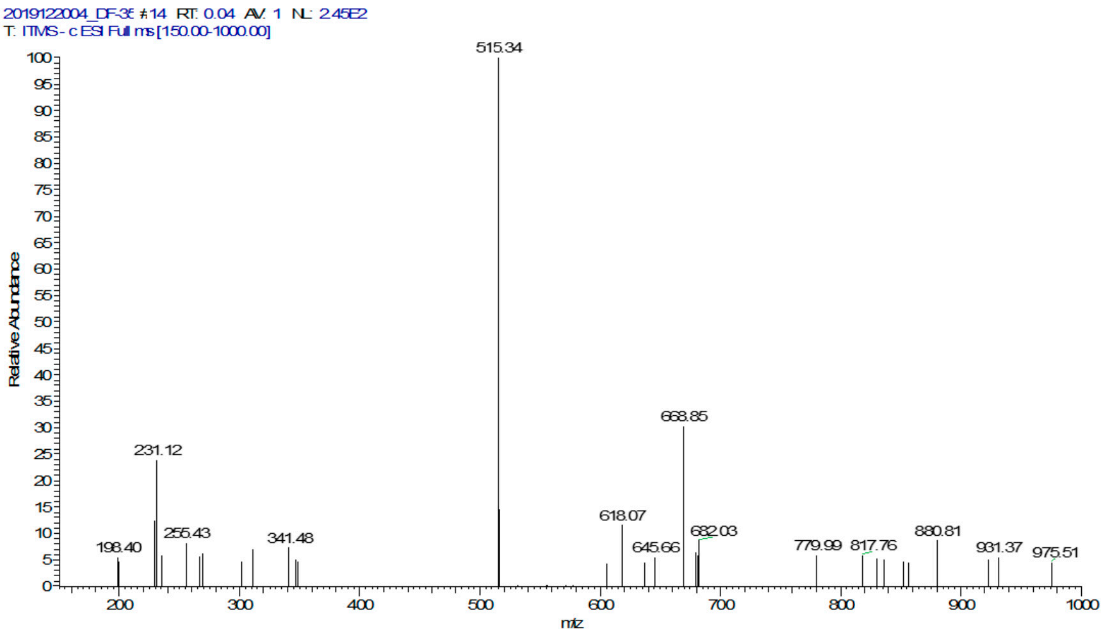


Mass spectrometry of **DF-36**

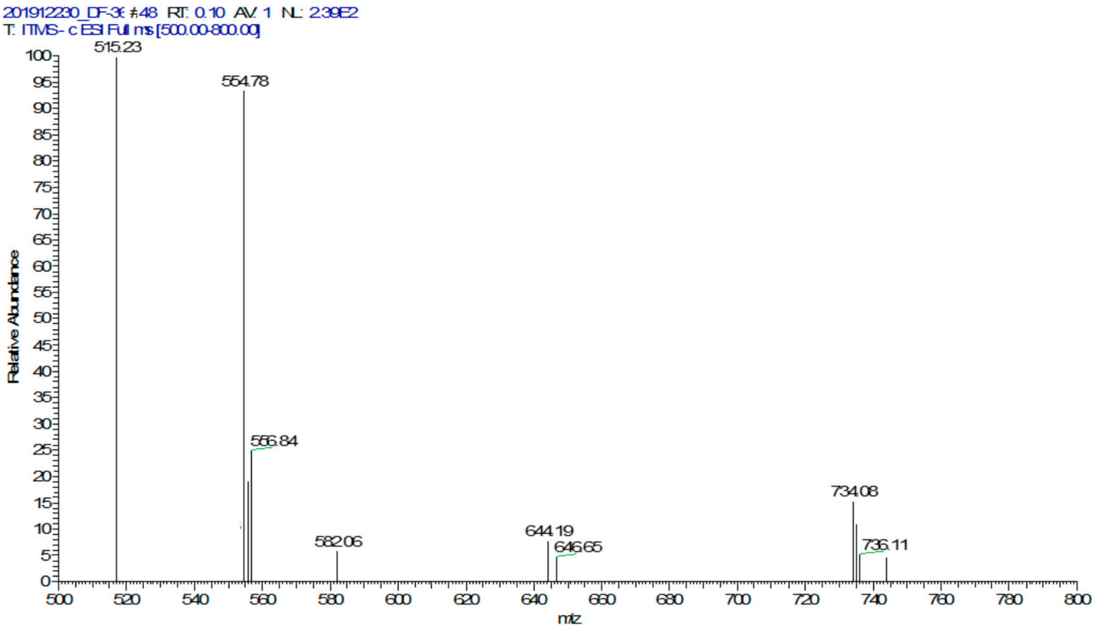

Mass spectrometry of **DF-47**

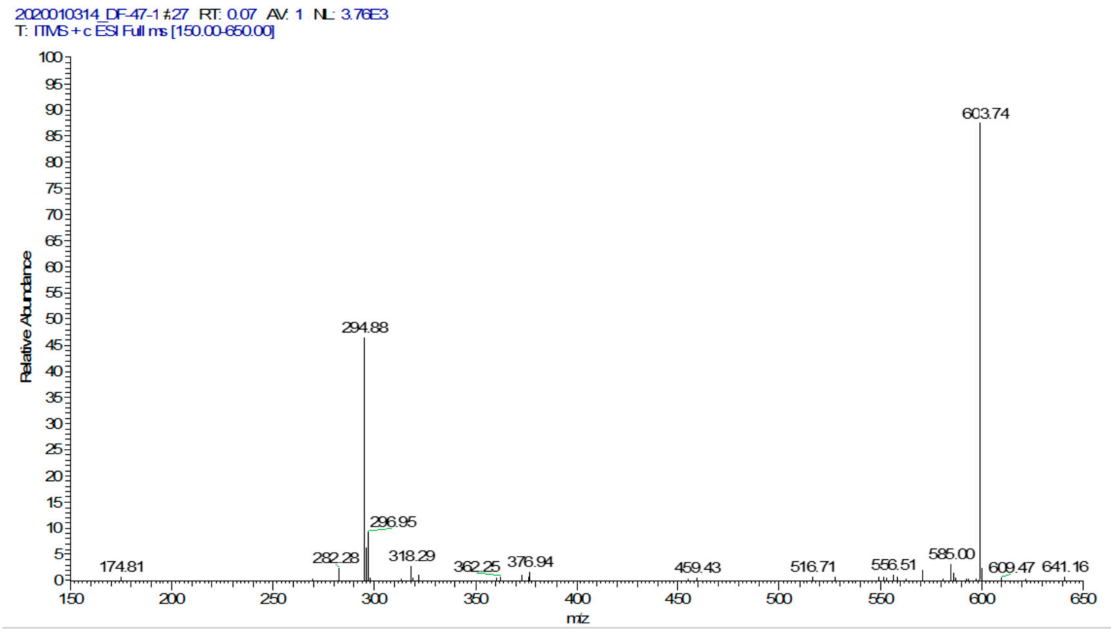


Mass spectrometry of **DF-51**

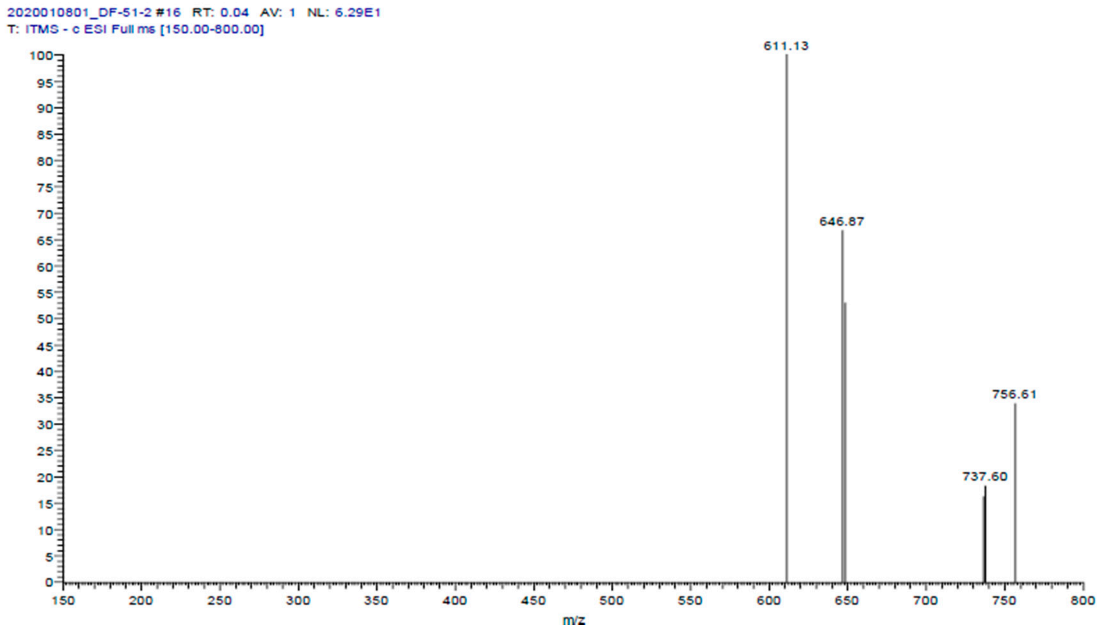

Mass spectrometry of **DF-57**

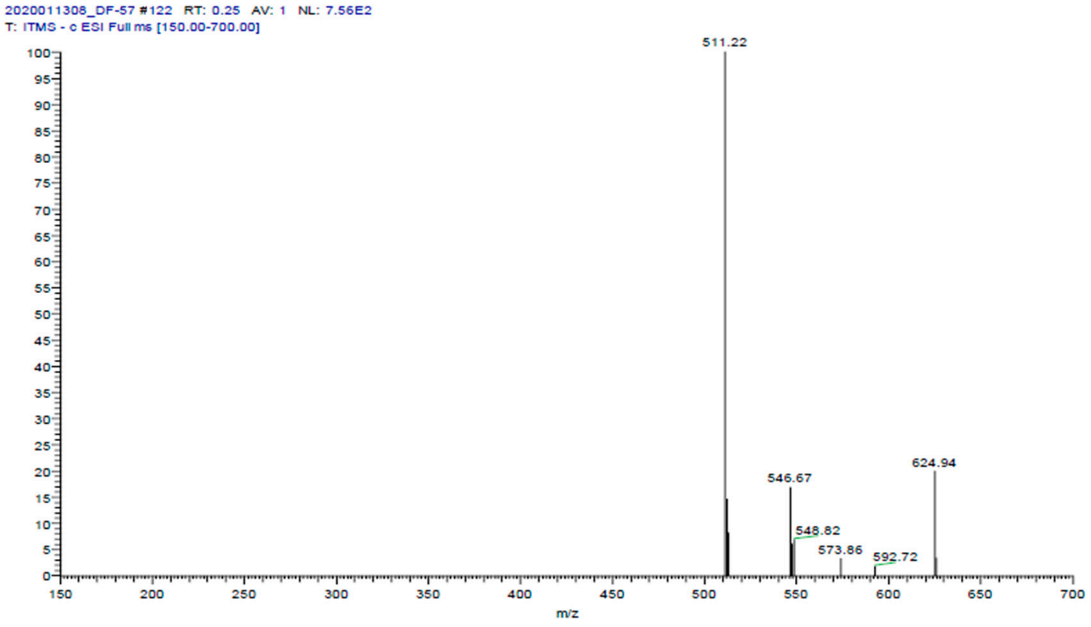


Mass spectrometry of **DF-63**

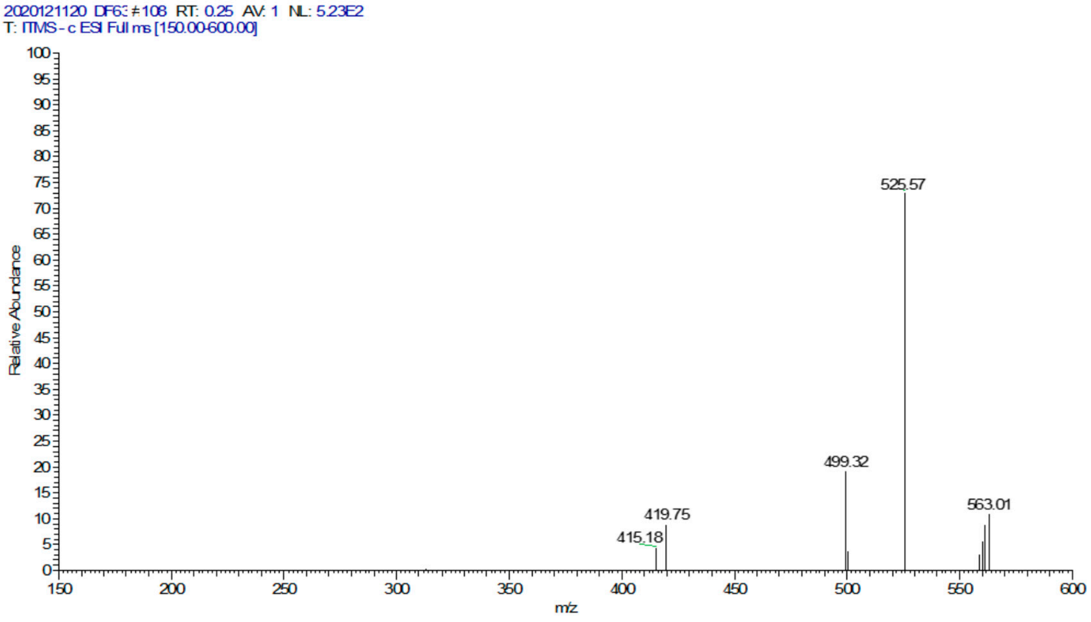

Mass spectrometry of **DF-64**

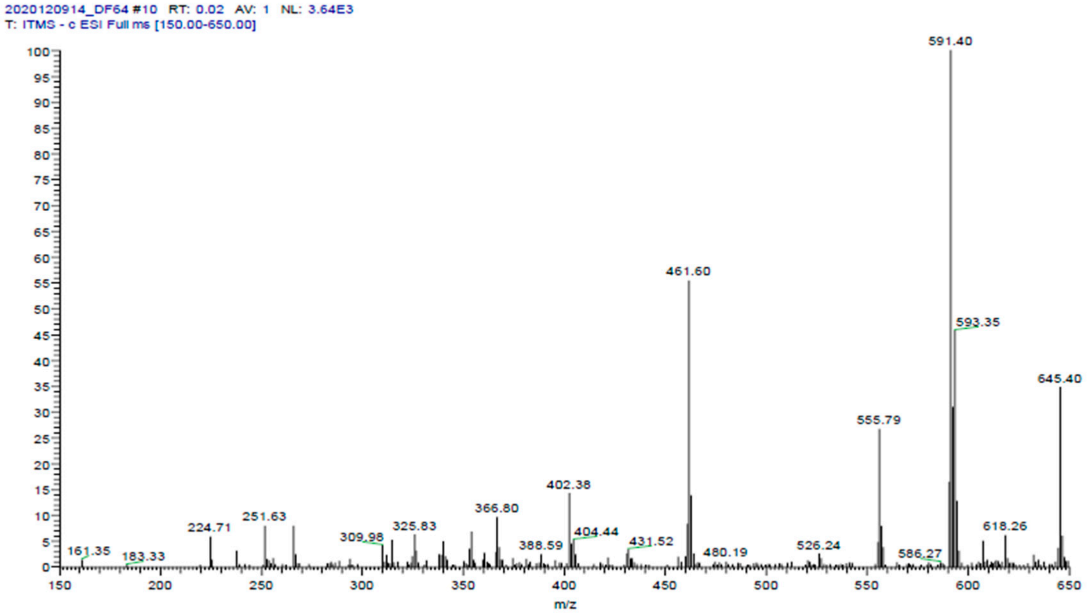


Mass spectrometry of **DF-67**

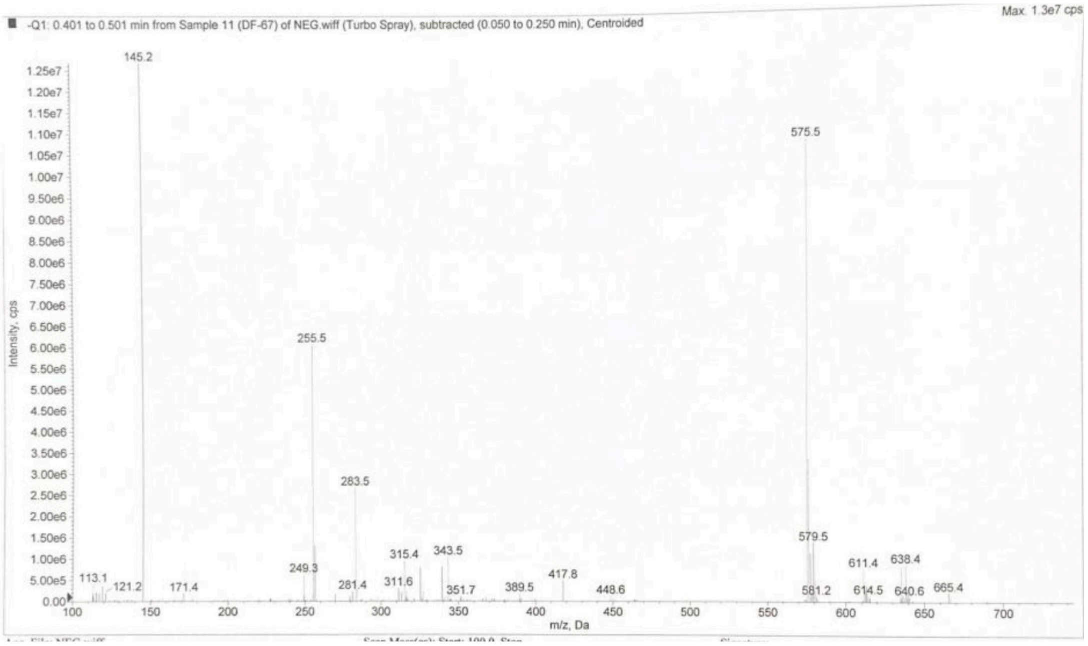

Mass spectrometry of **DF-68**

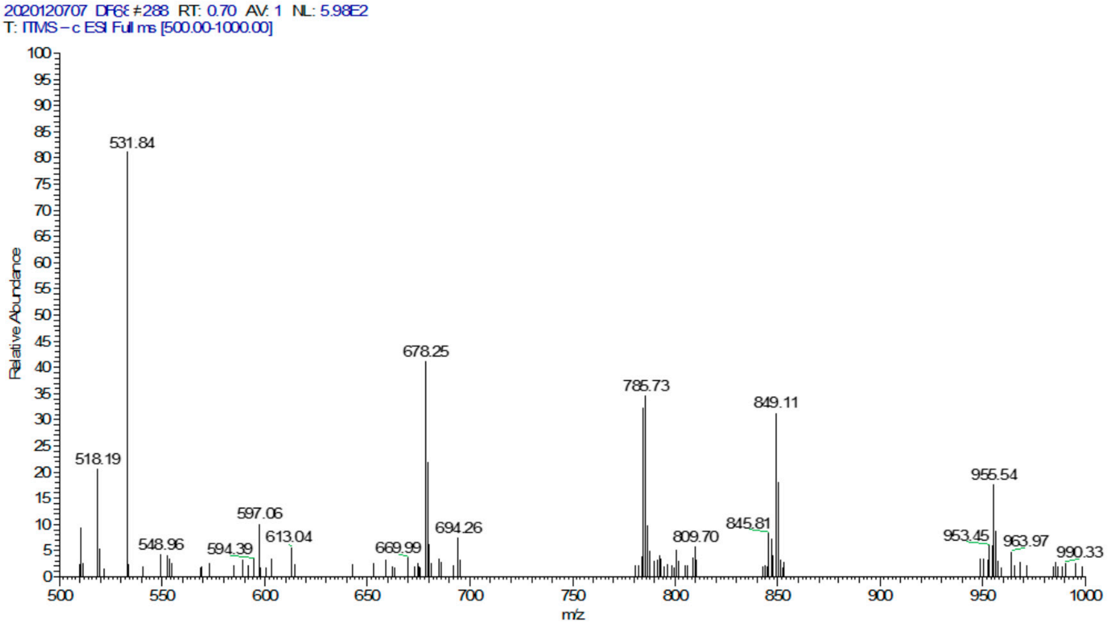


Mass spectrometry of **DF-69**

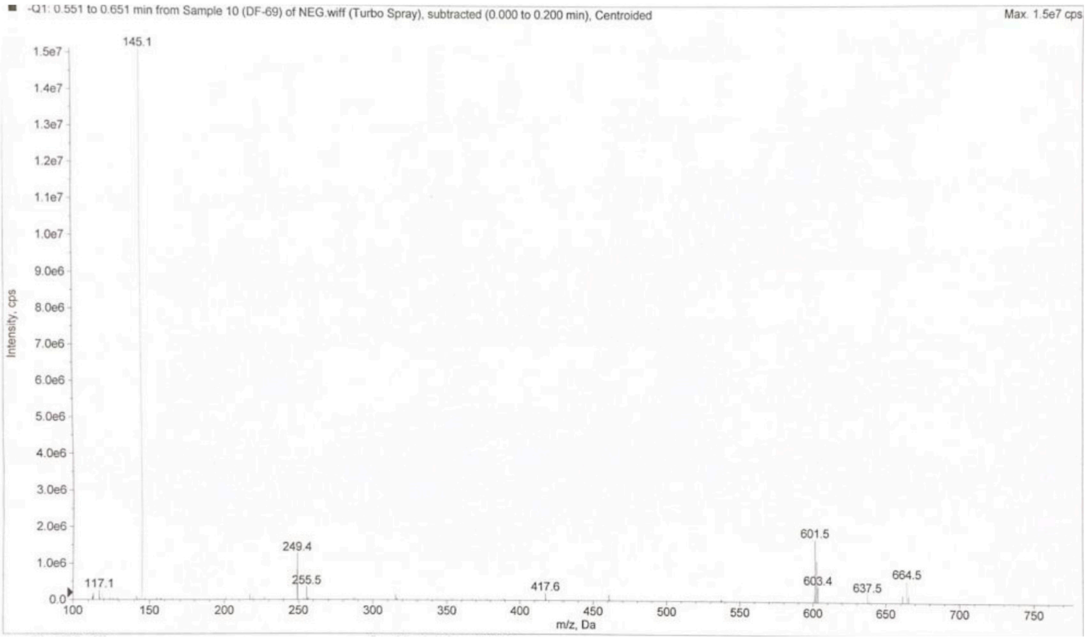

## HPLC traces of representative compounds

HPLC trace of compound **DF-47**

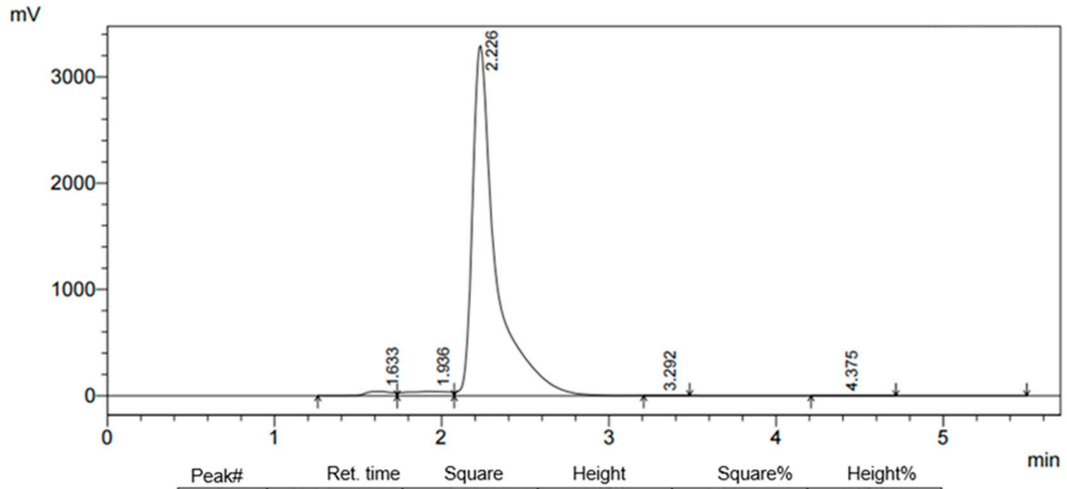


| Peak# | Ret. time | Square | Height | Square% | Height% |
|---|---|---|---|---|---|
| 1 | 1. 633 | 441599 | 39892 | 1. 329 | 1. 182 |
| 2 | 1. 936 | 736808 | 40884 | 2. 217 | 1. 212 |
| 3 | 2. 226 | 32050816 | 3292912 | 96. 436 | 97. 584 |
| 4 | 3. 292 | 4855 | 639 | 0. 015 | 0. 019 |
| 5 | 4. 375 | 1254 | 103 | 0. 004 | 0. 003 |
| Total | | 33235332 | 3374430 | 100. 000 | 100. 000 |

HPLC trace of compound **DF-51**

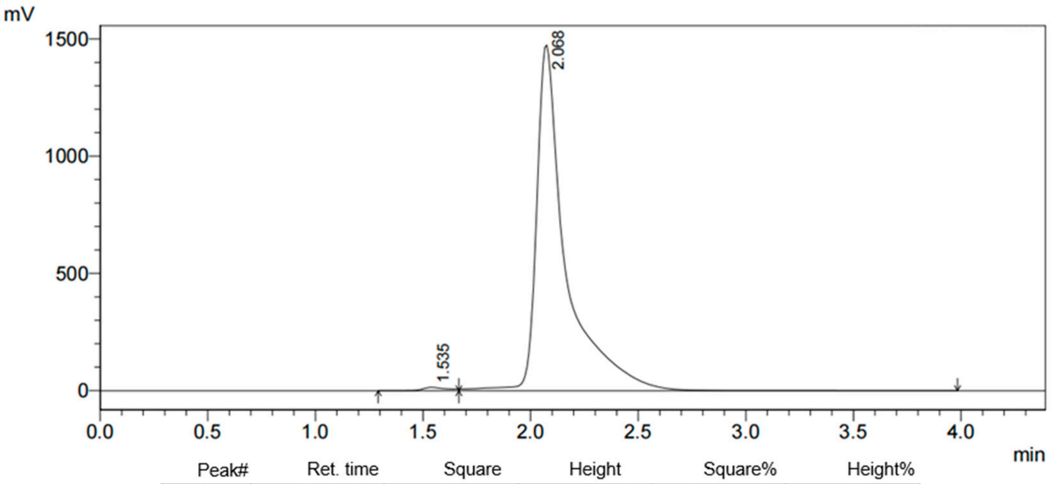


| Peak# | Ret. time | Square | Height | Square% | Height% |
|---|---|---|---|---|---|
| 1 | 1. 535 | 116456 | 14285 | 0. 826 | 0. 959 |
| 2 | 2. 068 | 13980431 | 1474763 | 99. 174 | 99. 041 |
| Total | | 14096887 | 1489049 | 100. 000 | 100. 000 |

**Full list of in-house metal chelator library compounds** (118 in total)

| No. | Structure | No. | Structure |
|---|---|---|---|
| 7a | 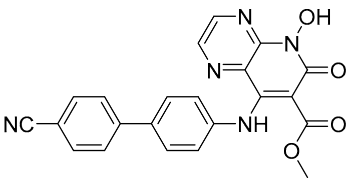 | 7b | 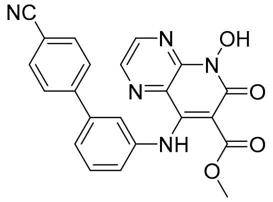 |
| 7c | 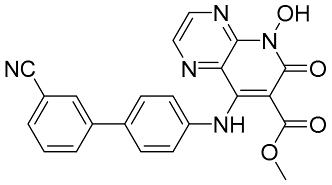 | 7d | 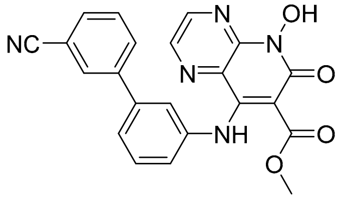 |
| 7e | 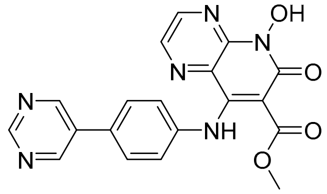 | 7f | 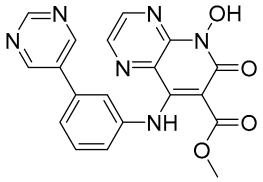 |
| 7g | 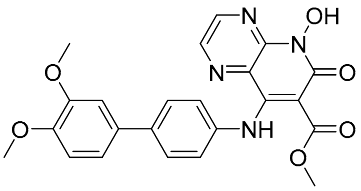 | 7h | 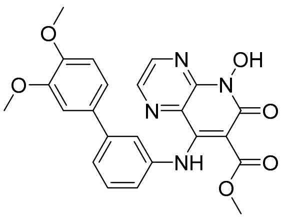 |
| 7i | 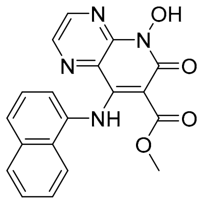 | 7j | 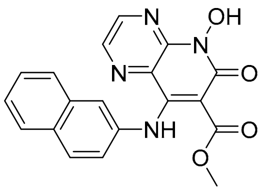 |
| 7k | 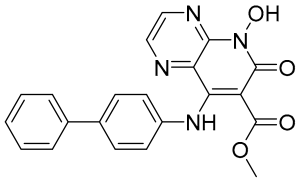 | - | - |
| I-1 | 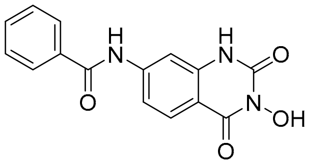 | I-2 | 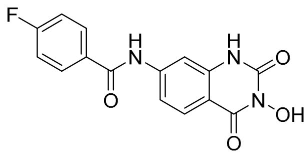 |
| I-3 | 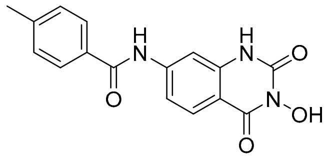 | I-4 | 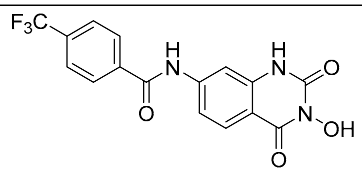 |

| | | | |
|---|---|---|---|
| I-5 | 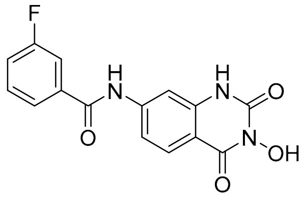 | I-6 | 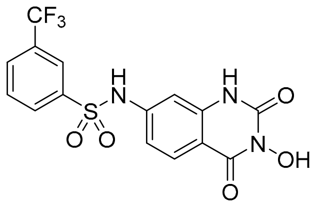 |
| I-7 | 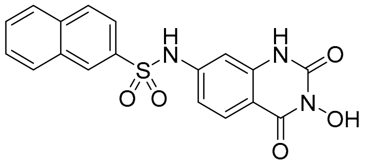 | I-8 | 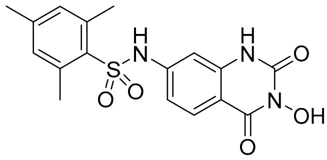 |
| I-9 | 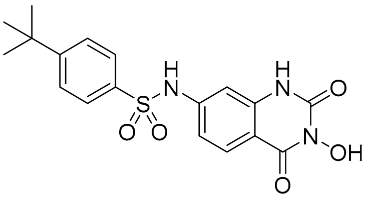 | I-10 | 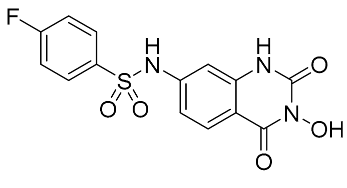 |
| I-11 | 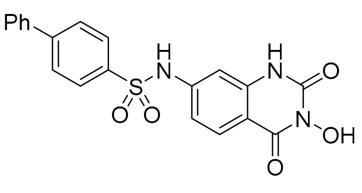 | I-12 | 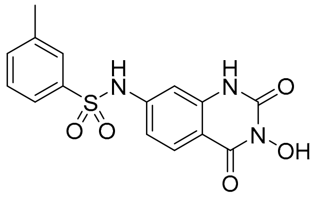 |
| II-1 | 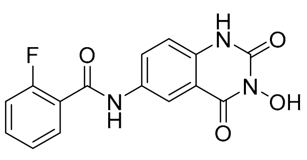 | II-2 | 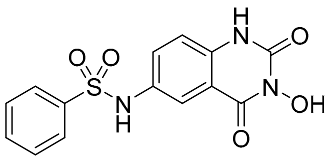 |
| II-3 | 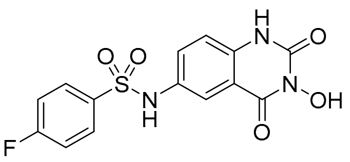 | II-4 | 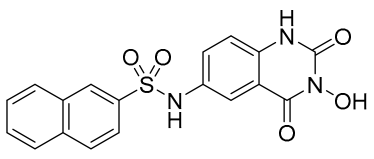 |
| II-5 | 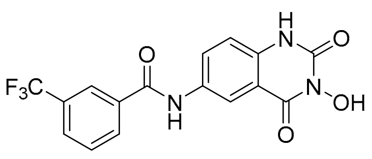 | | |
| III-1 | 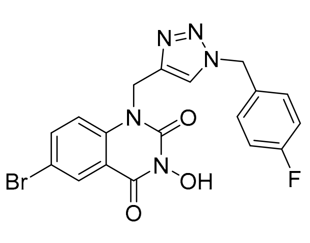 | III-2 | 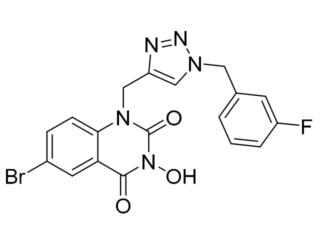 |
| III-3 | 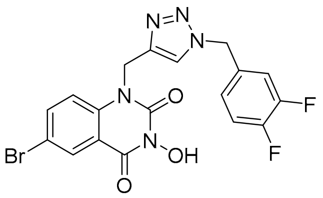 | III-4 | 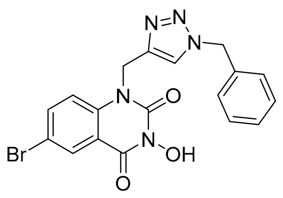 |

| | | | |
|---|---|---|---|
| III-5 | 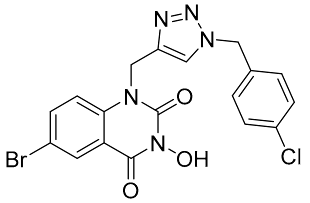 | III-6 | 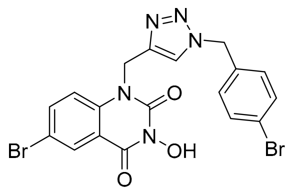 |
| III-7 | 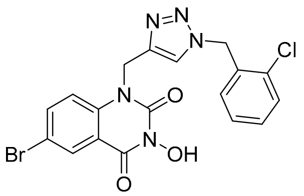 | III-8 | 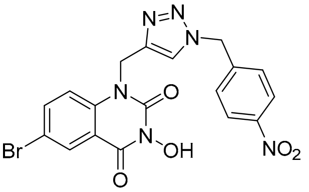 |
| III-9 | 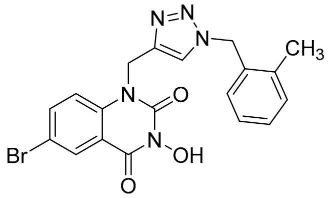 | III-10 | 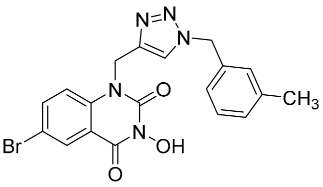 |
| IVA-1 | 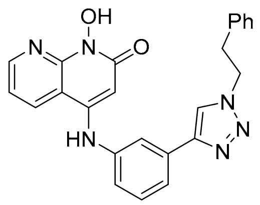 | IVA-2 | 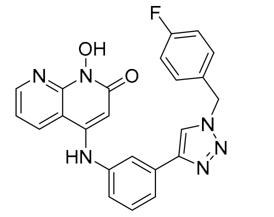 |
| IVA-3 | 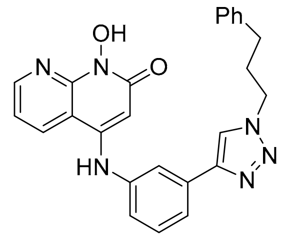 | IVA-4 | 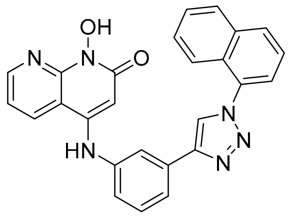 |
| IVA-5 | 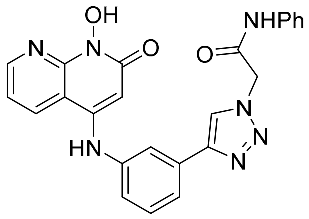 | IVA-6 | 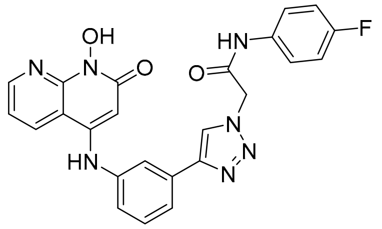 |
| IVA-7 | 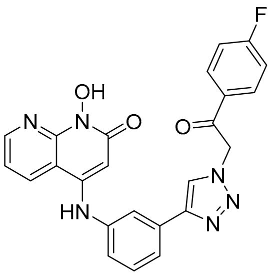 | IVA-8 | 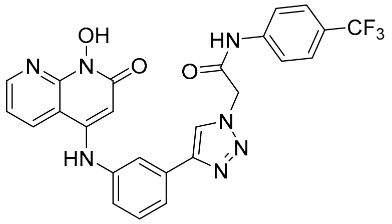 |

| | | | |
|---|---|---|---|
| IVB-1 | 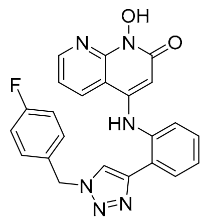 | IVB-2 | 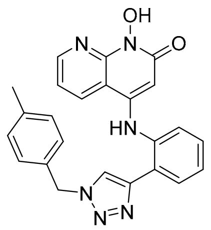 |
| V-1 | 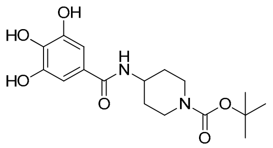 | V-2 | 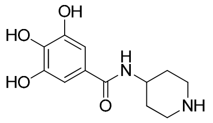 |
| V-3 | 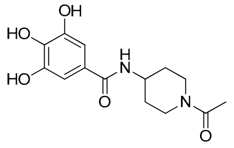 | V-4 | 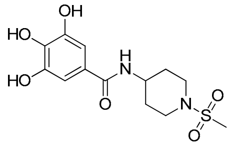 |
| V-5 | 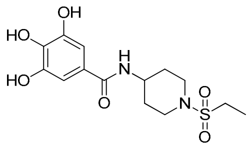 | V-6 | 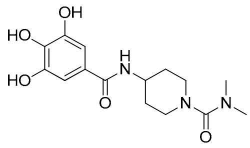 |
| V-7 | 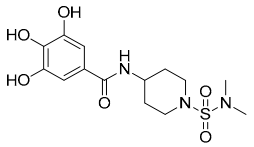 | V-8 | 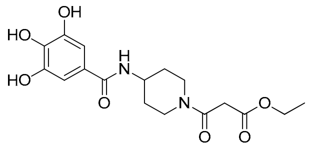 |
| V-9 | 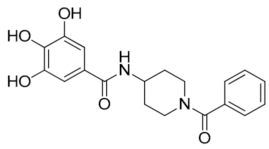 | V-10 | 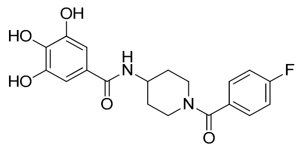 |
| V-11 | 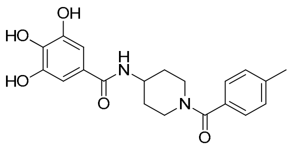 | V-12 | 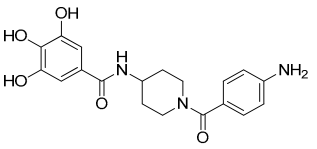 |
| V-13 | 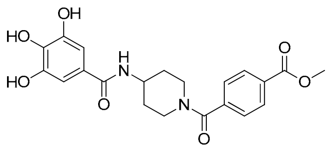 | V-14 | 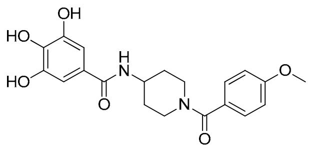 |
| V-15 | 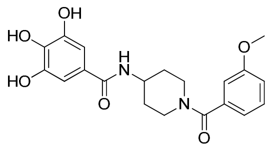 | V-16 | 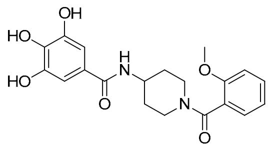 |
| V-17 | 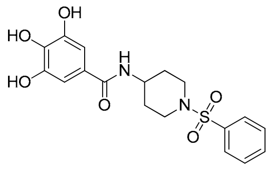 | V-18 | 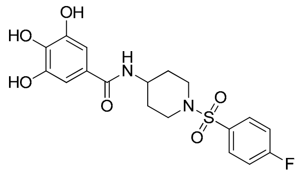 |

| | | | |
|---|---|---|---|
| V-19 | 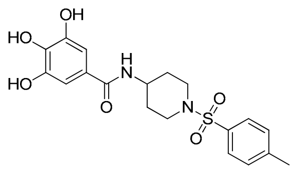 | V-20 | 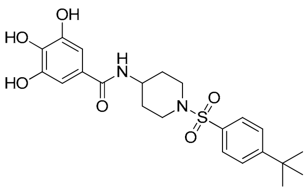 |
| V-21 | 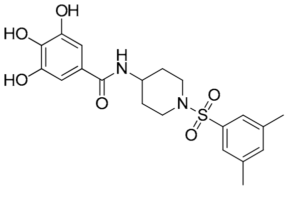 | V-22 | 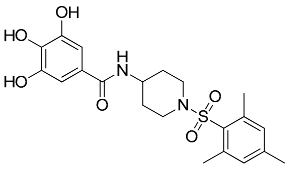 |
| V-23 | 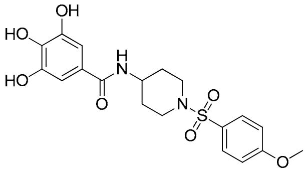 | V-24 | 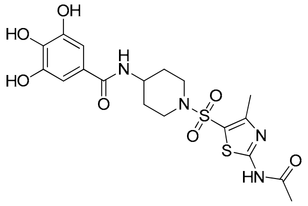 |
| VI-1 | 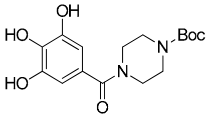 | VI-2 | 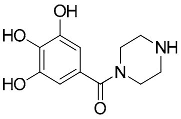 |
| VI-3 | 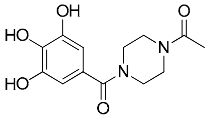 | VI-4 | 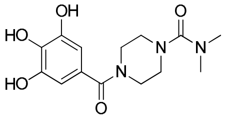 |
| VI-5 | 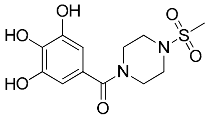 | VI-6 | 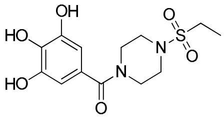 |
| VI-7 | 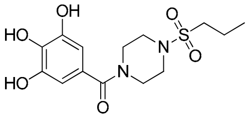 | VI-8 | 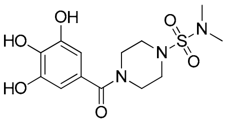 |
| VI-9 | 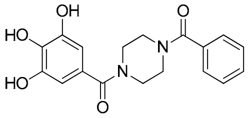 | VI-10 | 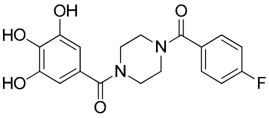 |
| VI-11 | 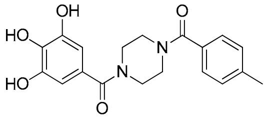 | VI-12 | 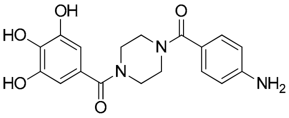 |
| VI-13 | 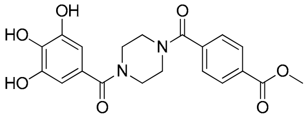 | VI-14 | 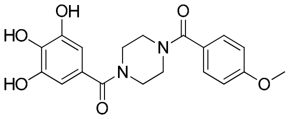 |
| VI-15 | 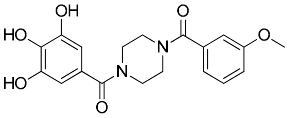 | VI-16 | 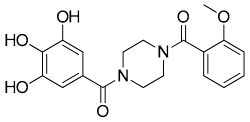 |
| VI-17 | 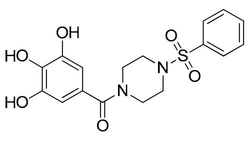 | VI-18 | 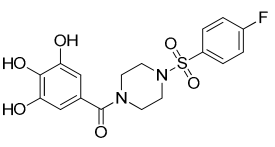 |

| | | | |
|---|---|---|---|
| VI-19 | 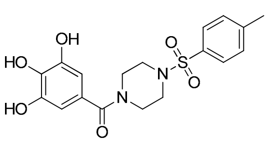 | VI-20 | 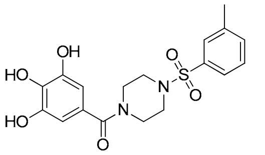 |
| VI-21 | 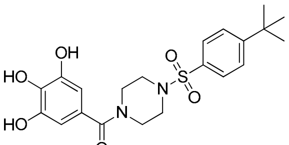 | VI-22 | 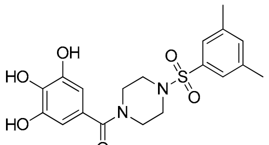 |
| VI-23 | 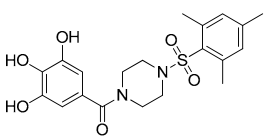 | VI-24 | 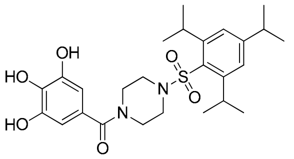 |
| VI-25 | 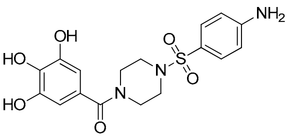 | VI-26 | 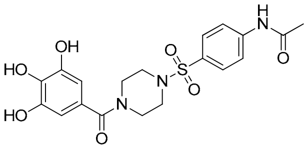 |
| DF-27 | 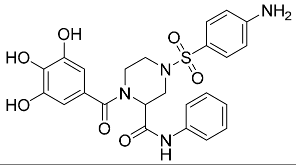 | DF-30 | 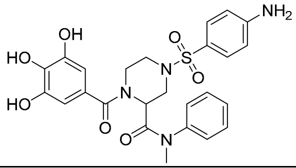 |
| DF-31 | 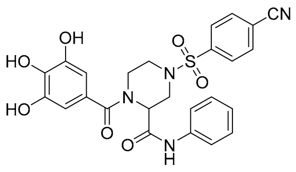 | DF-32 | 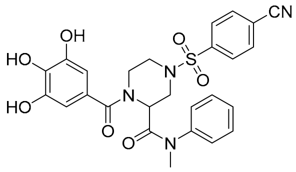 |
| DF-35 | 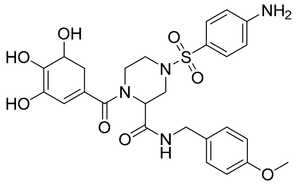 | DF-36 | 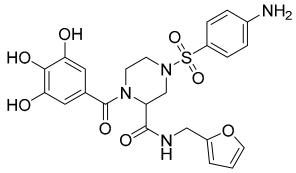 |
| DF-38 | 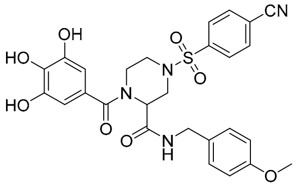 | DF-46 | 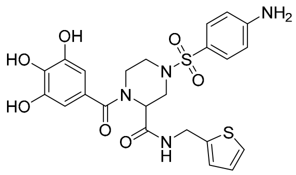 |
| DF-47 | 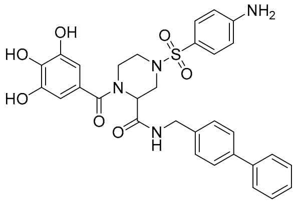 | DF-51 | 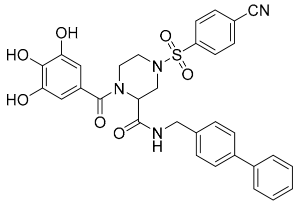 |

| | | | |
|---|---|---|---|
| DF-52 | 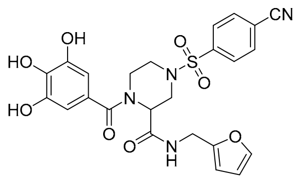 | DF-53 | 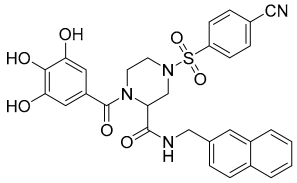 |
| DF-57 | 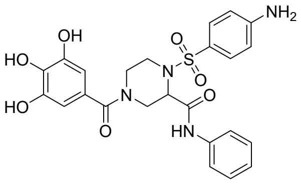 | DF-63 | 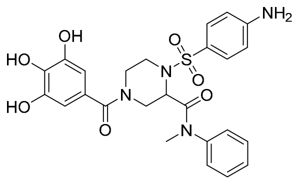 |
| DF-64 | 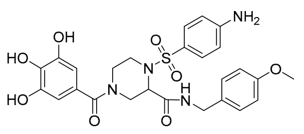 | DF-66 | 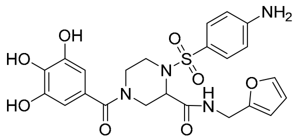 |
| DF-67 | 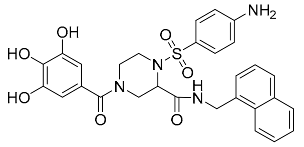 | DF-68 | 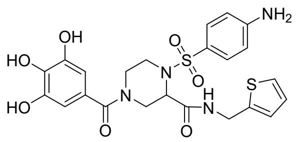 |
| DF-69 | 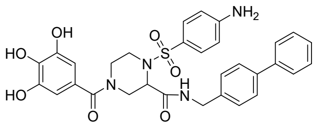 | DF-71 | 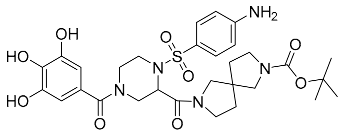 |
